# Acoustic change responses to amplitude modulation: a method to quantify cortical temporal processing and hemispheric asymmetry

**DOI:** 10.3389/fnins.2015.00038

**Published:** 2015-02-11

**Authors:** Ji Hye Han, Andrew Dimitrijevic

**Affiliations:** Communication Sciences Research Center, Cincinnati Children's Hospital Medical CenterCincinnati, OH, USA

**Keywords:** temporal processing, N1 auditory evoked potentials, temporal modulation transfer function, cortex, LORETA, dipole, source analysis

## Abstract

**Objective:** Sound modulation is a critical temporal cue for the perception of speech and environmental sounds. To examine auditory cortical responses to sound modulation, we developed an acoustic change stimulus involving amplitude modulation (AM) of ongoing noise. The AM transitions in this stimulus evoked an acoustic change complex (ACC) that was examined parametrically in terms of rate and depth of modulation and hemispheric symmetry.

**Methods:** Auditory cortical potentials were recorded from 64 scalp electrodes during passive listening in two conditions: (1) ACC from white noise to 4, 40, 300 Hz AM, with varying AM depths of 100, 50, 25% lasting 1 s and (2) 1 s AM noise bursts at the same modulation rate. Behavioral measures included AM detection from an attend ACC condition and AM depth thresholds (i.e., a temporal modulation transfer function, TMTF).

**Results:** The N1 response of the ACC was large to 4 and 40 Hz and small to the 300 Hz AM. In contrast, the opposite pattern was observed with bursts of AM showing larger responses with increases in AM rate. Brain source modeling showed significant hemispheric asymmetry such that 4 and 40 Hz ACC responses were dominated by right and left hemispheres respectively.

**Conclusion:** N1 responses to the ACC resembled a low pass filter shape similar to a behavioral TMTF. In the ACC paradigm, the only stimulus parameter that changes is AM and therefore the N1 response provides an index for this AM change. In contrast, an AM burst stimulus contains both AM and level changes and is likely dominated by the rise time of the stimulus. The hemispheric differences are consistent with the asymmetric sampling in time hypothesis suggesting that the different hemispheres preferentially sample acoustic time across different time windows.

**Significance:** The ACC provides a novel approach to studying temporal processing at the level of cortex and provides further evidence of hemispheric specialization for fast and slow stimuli.

## Introduction

Timing information is crucial for all aspects of hearing including sound detection, sound localization, and sound identification. Our ability to understand spoken language is heavily dependent on the information extracted from the temporal envelope of speech (Shannon et al., 1995; Smith et al., 2002). Insights to the importance of temporal processing for speech understanding can be gained from the study of disorders known to affect temporal processing such as auditory neuropathy (AN; Starr et al., 1996) which results in reduced speech perception ability, or by the restoration of speech perception ability through mostly temporal processing via a cochlear implant (CI; Shannon et al., 1995; Fu, 2002).

In this report we describe the use of the acoustic change complex (ACC) to quantify temporal processing ability in normal hearing adults. The ACC stimulus paradigm differs from “traditional” cortical evoked potential stimuli (e.g., a tone burst) in that the ACC N1/P2 response is elicited by a “change” in a single acoustic parameter, whereas a tone burst contains multiple stimulus parameter changes such as frequency and intensity. We previously reported the use of the ACC in a continuous tone paradigm where the frequency of the tone changes (Dimitrijevic et al., 2008). In that study, progressively smaller stimulus frequency changes elicited smaller ACC N1/P2 responses. Near subject's frequency discrimination thresholds, the ACC N1/P2 response became less detectable. In this study the “change” is amplitude modulation (AM) and the continuous baseline stimulus is stationary white noise. We reasoned that large, suprathreshold AM depths would elicit a large ACC N1/P2 response while smaller depths of AM that approach AM detection thresholds would elicit smaller ACC N1/P2 responses. We also manipulated the AM rate and reasoned that a temporal modulation transfer function (TMTF) could be obtained using ACC N1/P2 responses.

Temporal processing ability is often behaviorally quantified using the TMTF and measures the ability to detect AM as a function of AM rate (TMTFs; Viemeister, 1979). A typical TMTF is usually characterized by a low-pass filter shape such that there is low AM detection threshold at low frequencies and elevated AM detection thresholds above 50 Hz. One of the objectives of this study is to obtain an objective measure of TMTFs based on cortical evoked potentials that could potentially be used to assess temporal processing ability in people with CIs or AN.

It is not surprising that altered TMTFs are related to speech perception deficits given that the speech signal innately has AM. Rosen (1992) described the AM characteristics of speech illustrating that different aspects of speech signal are characterized by different AM rates. Syllables are typically in the 3–7 Hz range, while phonemic information in the 12–50 Hz range. How the brain decodes the acoustic speech signal to form speech perception is an active area of research. One hypothesis put forth by Poeppel (2003) is the Asymmetric Sampling in Time (AST) hypothesis which posits that the different hemispheres “sample” acoustic input at different rates. Left auditory areas preferentially process information at short time scales (~20 to 40 ms/25 to 50 Hz) while the right hemisphere extracts information at rapid time scales (~150 to 250 ms/4 to 7 Hz). Multiple lines of evidence for hemispheric asymmetry of acoustic processing have been observed using electrophysiological measures (Abrams et al., 2008; Dimitrijevic et al., 2008; Okamoto et al., 2009; Poelmans et al., 2012), functional neuroimaging (Zatorre and Belin, 2001), and optical neuroimaging (Telkemeyer et al., 2009).

The use of auditory evoked potentials to examine temporal processing has been the topic of a recent review by Picton (2013). In that review, it was noted that temporal resolution is often assessed using AM stimuli evoking an auditory steady-state response (ASSR), gap detection or recognition of single vs. double stimuli. Purcell et al. (2004) described a method of assessing temporal processing by presenting an AM stimulus that varied in modulation rate as a function of time. The brainstem ASSRs (above 70 Hz AM rate) were significantly correlated to behavioral measures of gap and AM detection. Cortical evoked potentials to gaps in AM stimuli have been found to be related to behavioral temporal processing differences between young and elderly (Ross et al., 2010) as well as in a later study, detecting brief gaps in continuous noise evoked cortical P2-N2 responses (Harris et al., 2012). Michalewski et al. (2005) reported a close relationship between gap detection thresholds and evoked potentials in normal-hearing and AN participants. In a more recent study, Miyazaki et al. (2013) developed an MEG paradigm using two pure tones differing in frequency to assess temporal processing in normal hearing participants. The evoked response (transient and ASSR) was generated to the beat and by adjusting beat frequency (2 to 60 Hz), and a TMTF could be obtained. In this study we were motivated to find a cortical correlate of temporal processing using AM stimuli in a paradigm that resembles a behaviorally determined TMTF. We reasoned that N1 responses to a “change” from white noise to AM of various depths and rates would resemble a behavioral TMTF. We did not want to use ASSRs to generate a “neural TMTF” for two main reasons: (1) the ASSR measures the ability of the nervous system to “follow” a long duration AM stimulus whereas in a behavioral TMTF paradigm, the subject is asked to discriminate between modulation and no modulation. Although the ASSR at different rates and different depths may be related to behavioral AM discrimination, the overall tasks (behavioral vs. electrophysiological) are very different. We therefore reasoned that the ACC N1 response (noise to AM) would be closely related to behavioral measures of AM discrimination. (2) The dominant generator of the ASSR differs as a function of the AM rate with rates above 70 Hz dominated by brainstem and below 70 Hz by cortex (reviewed in Dimitrijevic and Ross, 2008). Therefore, TMTFs derived from ASSRs covering modulation rates typically found in psychoacoustic experiments (2 Hz to ~2000 Hz) would represent activity from different neural generators. For these reasons we decided to employ the N1-change paradigm, also referred to as an ACC that would index the brain's response to detect modulation change from non-modulated white noise. Although change responses have been reported in the literature since the 1960's and 1970's (Spoor et al., 1969; Jerger and Jerger, 1970; McCandless and Rose, 1970; Arlinger et al., 1976; Kohn et al., 1978), the past decade has recently witnessed a resurgence in this type of evoked potential. The ACC is an attractive paradigm for studying the electrophysiology of psychoacoustic phenomena because only one acoustic stimulus parameter can be manipulated. For example, a tone burst contains both intensity and frequency parameter changes (i.e., silence to sound) whereas a change paradigm would have a continuous tone with frequency change resulting in only one parameter change (i.e., frequency). Previous work has shown that the ACC can be elicited using pitch/spectral changes (Krumbholz et al., 2003; Dimitrijevic et al., 2008; Okamoto et al., 2009, 2012; Pratt et al., 2009; He et al., 2012; Bidelman and Grall, 2014); intensity changes (Harris et al., 2007; Dimitrijevic et al., 2009; Okamoto et al., 2009, 2012; He et al., 2012), speech changes (Ostroff et al., 1998; Martin and Boothroyd, 1999, 2000; Tremblay et al., 2003; Edmonds et al., 2010; Martin et al., 2010; Dimitrijevic et al., 2013), interaural coherence (Chait et al., 2006) and “order to disorder” changes, i.e., constant tone to random tone bursts (Chait et al., 2007). To our knowledge two studies have used an AM change paradigm (Okamoto et al., 2009, 2012). In those two studies the authors were concerned with cortical spectral and temporal processing. Their “change stimulus” was (40 Hz AM) utilizing pure tone carriers and the AM (temporal) change was contrasted with a spectral change. In both studies, the temporal (AM) change stimulus evoked greater responses in the left hemisphere whereas the spectral had greater activation of the right hemisphere. The current study differs in that the AM rates were chosen to be representative of frequencies used in behavioral TMTFs and the “carrier” in this study uses white noise since this is common in previous behavioral TMTF studies.

In the present study, we examined the use of N1 response to changes in AM. We reasoned that a single parameter change (i.e., AM) would elicit an N1 response that index the brain's ability to discriminate AM as a function of rate and therefore result in response profiles similar to behavioral TMTFs. Additionally, we hypothesized that low rate AM change would preferentially activate the right hemisphere while faster AM rates would preferentially activate the left hemisphere as predicted by the AST hypothesis (Poeppel, 2003).

## Material and methods

### Subjects

Ten (6 females, 4 males) subjects (mean age: 25.5 years, all self-reported right-handed) were recruited through Cincinnati Children's Hospital Medical Center, according to an Institutional Review Board (IRB) approved protocol. NH subjects were screened for audiometric hearing thresholds ≤20 dB HL at octave test frequencies from 250 to 8000 Hz, and had no neurological or psychological diseases. Participants were compensated for their participation. Informed consent was obtained from all participants.

### Stimuli

**Figure 1:**
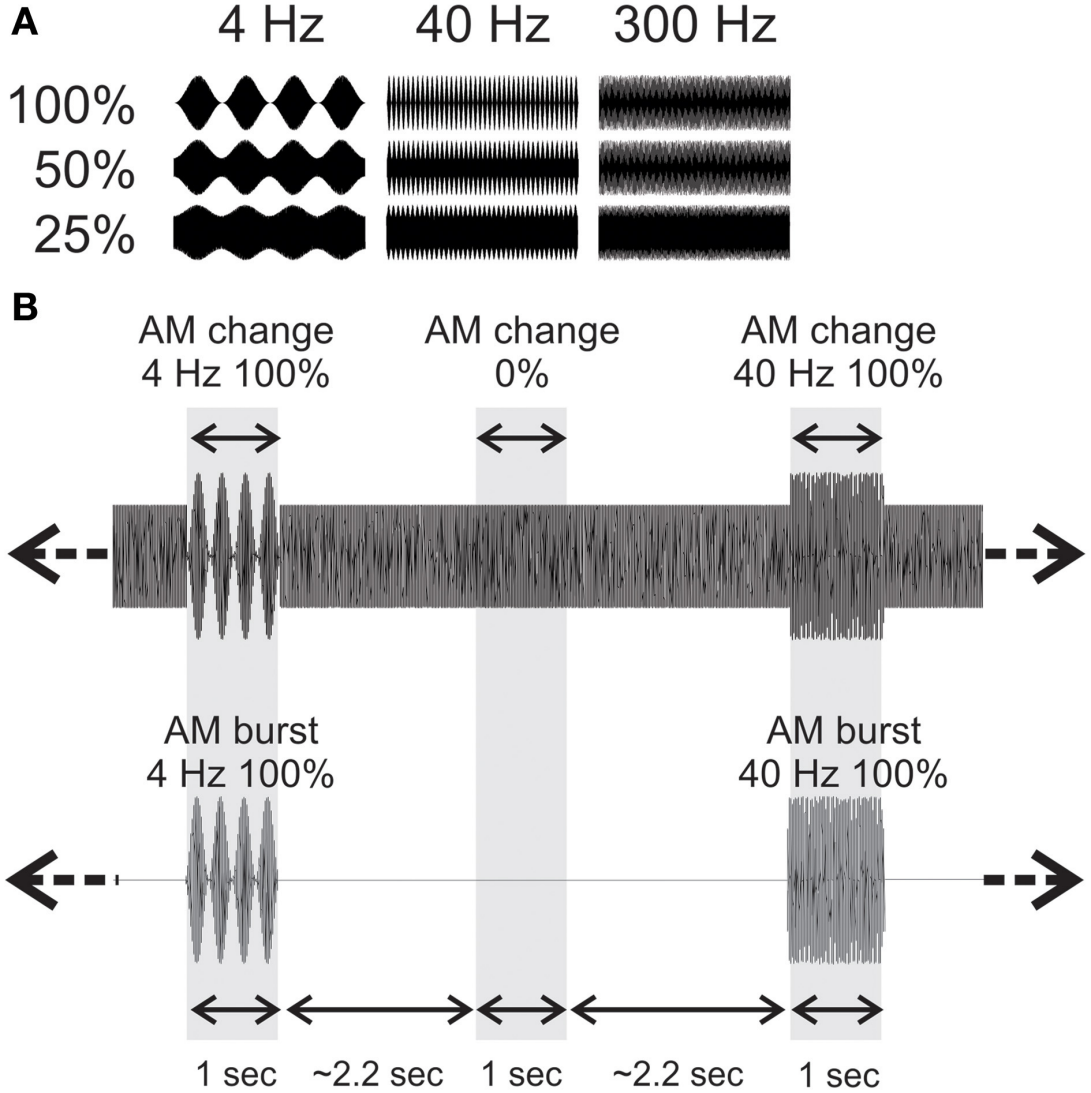
**Acoustic stimuli used**. **(A)** For the AM change stimuli, 3 AM rates (4, 40, and 300 Hz) and 3 modulation depths were used. The time scale is one second. **(B)** An example of an acoustic sequence in the two conditions. The top row shows a ~6 s sample of 4 Hz 100% AM change, and a 40 Hz 100% AM change with the corresponding AM burst stimuli below. Note that in order to reduce possible loudness cues from no modulation to AM, the rms of the AM was equated with the 1 s preceding noise.

Stimuli were constructed in Matlab using continuous white noise with occasional changes consisting of AM lasting 1 s occurring on average every 2.2 s (random inter-stimulus interval varying from 1.8 to 2.6 s) and lasting 1.0 s. Each AM change stimulus and baseline segment was generated from a novel sample of noise using the *rand* function in Matlab. The AM rates were 4, 40, and 300 Hz. The modulation depths were included: 100%, 50%, 25% and 0% (Figure 1A). In order to avoid differences in overall level that would occur when AM is introduced, the AM portion of the stimulus was multiplied by a factor that equated the root mean square of the preceding 1 s (no modulation). Figure 1B shows approximately 7 s of a sample stimulus that was used. The AM burst stimuli were generated using the identical process as described above. However, only 4, 40, and 300 Hz 100% AM were used and after the root mean square was adjusted for intensity, the preceding baseline noise was set to zero. This procedure created bursts of AM with the same amplitude as the AM change.

Stimuli were presented in free field, delivered through one speaker at 0° azimuth 1.5 m away from the subject. The sound intensity at the approximate head location of a typical listener was at 70 dB SPL. We chose to use free field sound delivery because we intend to use these stimuli in studies with people with cochlear implants. The stimuli were calibrated using a Brüel and Kjær (Investigator 2260) sound level meter set on A weighting and slow time weighting with a ½inch free field microphone. Note that free field presentation is not ideal for ERP recordings because it may be possible that the listener could alter the intensity of the sound coming to each ear by simply moving their head. However, we do not feel this would greatly affect our results because the distance between the speaker and the listener was always the same across participants and head movements were monitored throughout the experiment ensuring no extreme head movements occurred.

### Recordings

The electrophysiological data was collected using a 64-channel actiCHamp Brain Products recording system (Brain Products GmbH, Inc., Munich, Germany). An electrode cap was placed on the scalp with electrodes placed at equidistant locations, the infracerebral cap covers a larger area than what is typical in a 10–20 system (Hine and Debener, 2007). The reference channel was located at vertex (Cz) while the ground electrode was located on the midline 50% of the distance to nasion. A representation of the cap layout is shown in Figure S1. Continuous data were digitized at 1000 Hz and stored for offline analysis.

### Data processing

Electrophysiological data were analyzed using Brain Vision Analyzer ver. 2.0 (Brain Products GmbH, Inc., Munich, Germany). Data were high-pass filtered (0.01 Hz) to remove baseline drifts and down sampled to 512 Hz. Visual inspection of the data included removal of extreme stereotypical artifacts related to subject movement (exceeding 500 mV). Independent component analysis (ICA; Delorme and Makeig, 2004) as implemented in Brain Vision Analyzer (identical algorithm as EEGLAB; Delorme and Makeig, 2004) was applied in order to reduce ocular and cardiac artifacts. On average 3 independent components were removed per subject.

After ICA artifact reduction, the data were averaged referenced and segmented into epochs −200 to 1500 ms with the AM change stimulus occurring at 0 ms. The auditory N1 response was identified for further analysis. The N1 response is a composite brain potential made up of different overlapping components (e.g., N1a, N1b, and N1c). The reader is referred to Näätänen and Picton (1987) and Woods (1995) for reviews on the separate components of N1. The N1b responses were observed by pooling 3 electrodes in the frontal-central regions (FC; see Figure S1) and had peak latencies near 120 ms. N1c responses were observed at left and right temporal electrodes (LT and RT; see Figure S1) with negative peaks near 170 ms, note that these potentials are also referred to as T-complexes (Näätänen and Picton, 1987; Woods, 1995). The N1b and N1c amplitudes were calculated separately based on mean voltage over a 20 ms window centered on the peak waveform as identified in the grand mean data. Therefore, the same window was used for all subjects (see Chapter 4, Luck, 2005).

### Procedures

During the EEG recording, participants were seated in a sound-attenuated booth. Subjects participated in two conditions, a passive and attend condition. In the passive condition, subjects watched a silent, closed-captioned movie of their choice, and instructed to ignore the background sounds. A total of one hundred trials of each of the 9 AM change stimuli (i.e., three rates and three depths) were presented across four blocks (each with 25 trials). The total recording time was approximately 1.5 h and subjects were encouraged to take breaks between blocks. In the attend condition, the same nine sets of AM change stimuli were presented and subjects were instructed to press a button whenever they detected any “change” in the ongoing noise. The attend condition always occurred after the passive condition. For purposes of brevity, only the behavioral data (accuracy and reaction time) from the attend condition are presented this report and a comparison of the passive vs. attend data will be described in a future report.

In a subset of subjects (*n* = 6), the behavioral threshold for AM depth detection for 4, 40, and 300 Hz was performed in a separate task using a three interval forced choice with trial-by-trial feedback (Levitt, 1971). The task used three intervals of a 1 s long white noise stimuli, one of which had AM. The minimum depth of AM needed to identify the AM interval was recorded after 9 reversals.

### Source analysis

#### Dipole source modeling

Dipole source analysis was performed using BESA Research 6.0 and was similar to the procedure described by Hine and Debener (2007) for all individual subjects, for the 100% AM change conditions and AM bursts. Two symmetric regional sources were initially seeded in a location near Heschl's gyrus (Talairch coordinates: ±49.5, −17, 9). A 20 ms window centered around N1 based on the global field power was used in the dipole fit. The maximum of the tangential source was fit on the N1 peak and BESA automatically sets the radial orientation to be perpendicular to the tangential source. These two sources represent the N1b and N1c (or T-complex) respectively. Only these two orientations were considered given that the third orientation did not show systematic relationships with the stimulus. A goodness of fit (GOF) was assessed for each subject over the 20 ms window. Subjects showing an 80% or higher GOF were further analyzed. Grand mean source waveforms across all subjects were performed. In order to assess statistical differences between conditions, the mean current over the 20 ms window centered on the peak of the tangential or radial sources were assessed in each subject. Additionally, a lateralization index (LI) was computed using the mean source activity over the same 20 ms window for each source waveform peak using the following formula

LI=(left−right)/(left+right)

The LI can vary between −1 and +1 with positive values indicating a leftward bias, and negative values a rightward bias. Values exceeding ±0.2 are often considered lateralized (Seghier, 2008).

#### Distributed source modeling

Distributed source modeling was assessed using swLORETA in BESA Research 6.0. swLORETA is a variation of sLORETA that includes depth weighting. The sLORETA approach is one of many methods to estimate brain source activation from scalp recorded potentials. We chose swLORETA with two successive iterations because pilot data using noise burst stimuli showed the most consistent N1 activations near Heschl's gyrus. Although no “gold standard” exists for a standardized pipeline analysis for BESA inverse solutions, we opted for an approach guided by mean area measurements of cortical waveforms (see Chapter 4, Luck, 2005). The analysis occurred over the following steps: the first step involved swLORETA modeling over a 20 ms window around N1b (and N1c separately) for each subject. The latency was based on the grand mean ERP waveform for the particular condition. Therefore, swLORETA was performed over the same latency for each subject. Secondly, a customized Matlab program was used to average all the individual image files together. This averaged image file was considered the grand mean swLORETA source. The third step identified local maxima in the grand mean swLORETA source analysis outcome. Typically, maxima sources were found in left and right superior temporal cortex near Heschl's gyrus, and frontal locations. The fourth step used the Talairach coordinates of the left and right auditory areas (obtained from the left and right maxima based on the grand mean source described above) to create a “virtual source time activation.” In this step, swLORETA was evaluated again in individual subjects, but this time over a 0 to 500 ms time range. The swLORETA cortical activations at the previously determined Talariach coordinates (left and right auditory) were then extracted. We interpret this activation to represent the time course of activation of the auditory generator of N1b (or N1c). Grand averaged virtual source time activations were created for each condition for left and right auditory activation areas. Mean activation amplitudes (left and right separately) were evaluated for a 20 ms time window (i.e., the same window used in the first swLORETA analysis) and were subjected to further statistical evaluation.

Another source analysis procedure involved examining swLORETA activation differences in source space using BESA Statistics. Differences between conditions were assessed by first evaluating the individual swLORETA activations (see above) and then a paired *t*-test was performed to test for significant differences between conditions. All *p*-values reported are corrected for multiple comparisons using Monte-Carlo resampling techniques (Maris and Oostenveld, 2007). Clusters of voxels with *p*-values of less than 0.05 were considered significant.

### Statistical analysis

Repeated measures ANOVAs were used to assess statistical significance. Greenhouse-Geisser corrections were applied where appropriate. Details of the repeated measures ANOVA factors are given with the results. *Post-hoc* analyses were assessed using Tukey's Honest Significantly Difference test.

## Results

### Psychoacoustics

**Figure 2:**
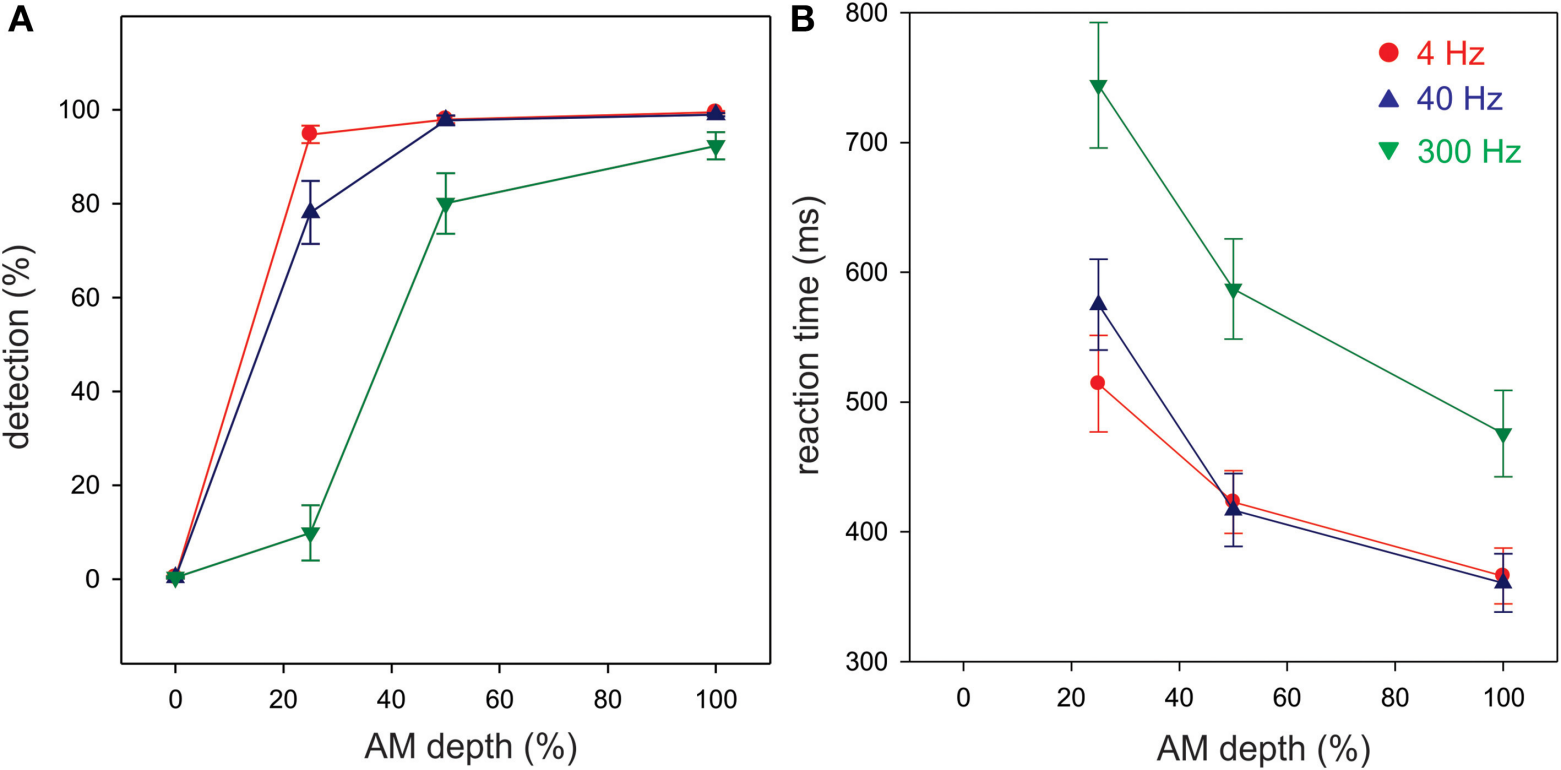
**Behavioral detection rates (A) and reaction times (B) in the attend ACC condition**. All three AM rates are overlaid. Each ACC stimulus was presented 100 times and the detection rates represent the proportion of the stimuli that were detected over 100 repetitions. Shown are the mean detection and reaction times across all 10 participants. Note that the 4 and 40 Hz AM stimuli were more detectable than the 300 Hz particularly with lower AM depths. The overall reaction time to 300 Hz AM was reduced compared to 4 and 40 Hz. Errors bars: standard error of the mean.

Detection and reaction times to button presses for AM change stimuli are presented in Figure 2. Most subjects were able to detect the 100% AM change stimuli for all three modulation rates. However, as the depth (%) of AM decreased, the detection rate also decreased. A repeated measures ANOVA revealed a significant AM rate × AM depth interaction [*F*_(6, 54)_ = 56.6; *p* < 0.001]. *Post-hoc* analysis revealed that at 25% and 50% AM depth, the detection rate for 300 Hz was lower than for 4 (*p* < 0.01) and for 40 Hz (*p* < 0.01). 4 Hz detection rate was greater than 40 Hz at 25% depth (*p* < 0.01).

Overall, the reaction times were longer for 300 Hz AM change compared to the other AM rates and were more delayed with smaller AM depths (Figure 2B). A repeated measures ANOVA revealed a significant AM rate × AM depth interaction [*F*_(4, 24)_ = 8.1, *p* < 0.001]. The reaction times for 300 Hz stimulus were longer compared to 4 (*p* < 0.01) and 40 Hz (*p* < 0.01). Also, the reaction times for 4 Hz and 40 Hz at 25% were significantly shorter than 4 Hz (*p* < 0.01) and 40 Hz (*p* < 0.001) at 100% depth. The reaction time for 300 Hz at 100% AM depth was significantly shorter than those for 300 Hz at 50% (*p* < 0.01) and 300 Hz at 25% (*p* < 0.001).

In a subset of six subjects, the behavioral AM depth thresholds as a function of AM rate were examined. The mean AM depth threshold for 4, 40, and 300 Hz was 13%, 11%, and 25% respectively. Note that this differed from the results presented above in that in these subjects the AM depth was varied adaptively to determine the minimum modulation depth needed to detect modulation whereas the above results are from the attend condition that had fixed AM depths. A repeated measures ANOVA revealed a main effect for AM rate [*F*_(2, 10)_ = 8.06, *p* < 0.001]. *Post-hoc* analysis showed that the AM threshold for 300 Hz was significantly higher than those for 4 Hz (*p* < 0.01) and for 40 Hz (*p* < 0.01). No significant difference in AM threshold was found between 4 and 40 Hz (*p* > 0.05).

### Cortical potentials

#### AM change: effects of AM rate

**Figure 3:**
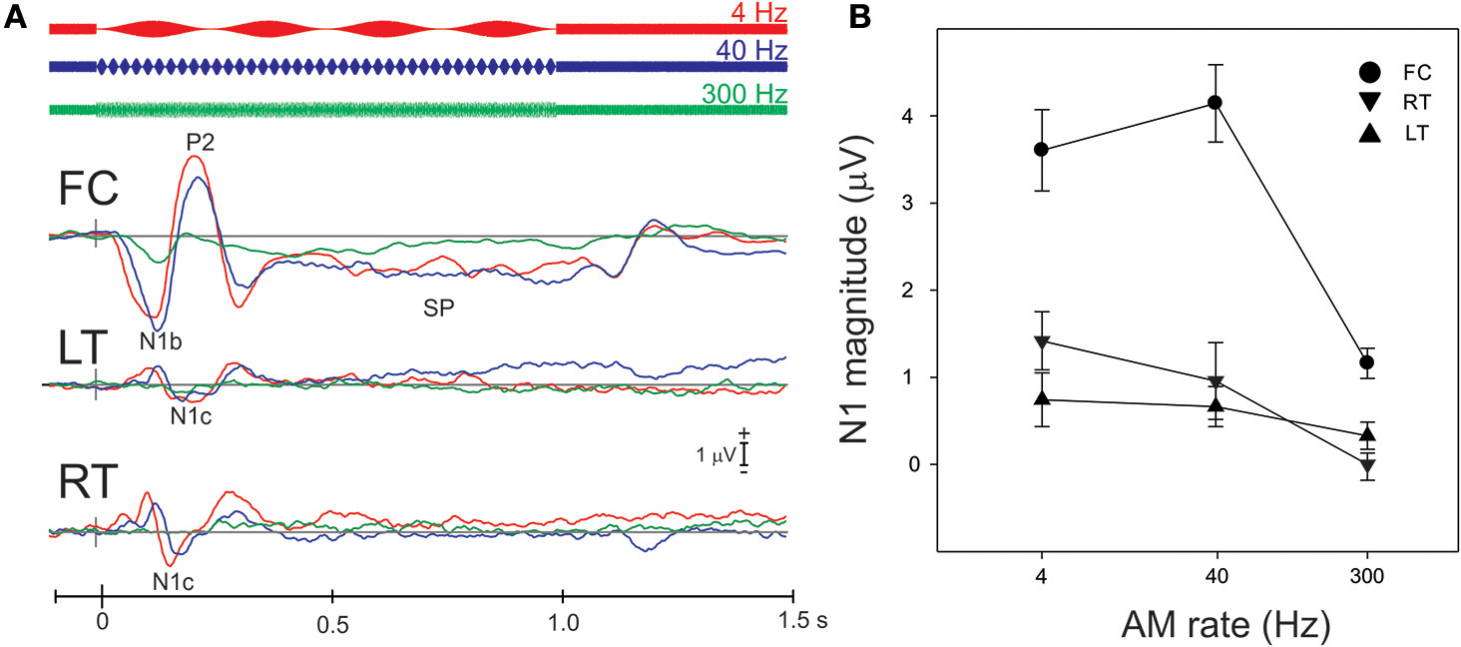
**Grand mean waveforms to the 100% AM change stimulus (A) and mean N1b and N1c amplitudes (B) are shown**. **(A)** Shows responses recorded at FC and the lateral (LT/RT) electrodes to the 4 (red), 40 (blue), 300 Hz (green) AM change stimulus. The response is characterized by N1, P2 response, followed by the sustained potential (SP) that lasts for the entire duration of the AM stimulus. An offset response (resumption of the noise from AM) is also seen. The N1b response recorded at FC electrodes is larger than the N1c (T-complex). **(B)** shows the mean averaged N1b/N1c amplitude as a function of AM rate over a 20 ms window across all subjects. Note that the latency for the window was based on the grand mean data **(A)** and the same latency window was used across all subjects. Errors bars: standard error of the mean.

Figure 3A shows the grand mean potentials at FC, LT, and RT electrodes to 100% AM change at 4, 40, and 300 Hz. The overall response is characterized by an N1b response occurring close to 120 ms after the onset of stimulus, followed by the sustained potential (SP) and an “off response.” The N1c responses (T-complex) were apparent at RT and LT electrodes and occurred at latencies near 170 ms. This report will focus on the N1b and N1c response.

**Figure 4:**
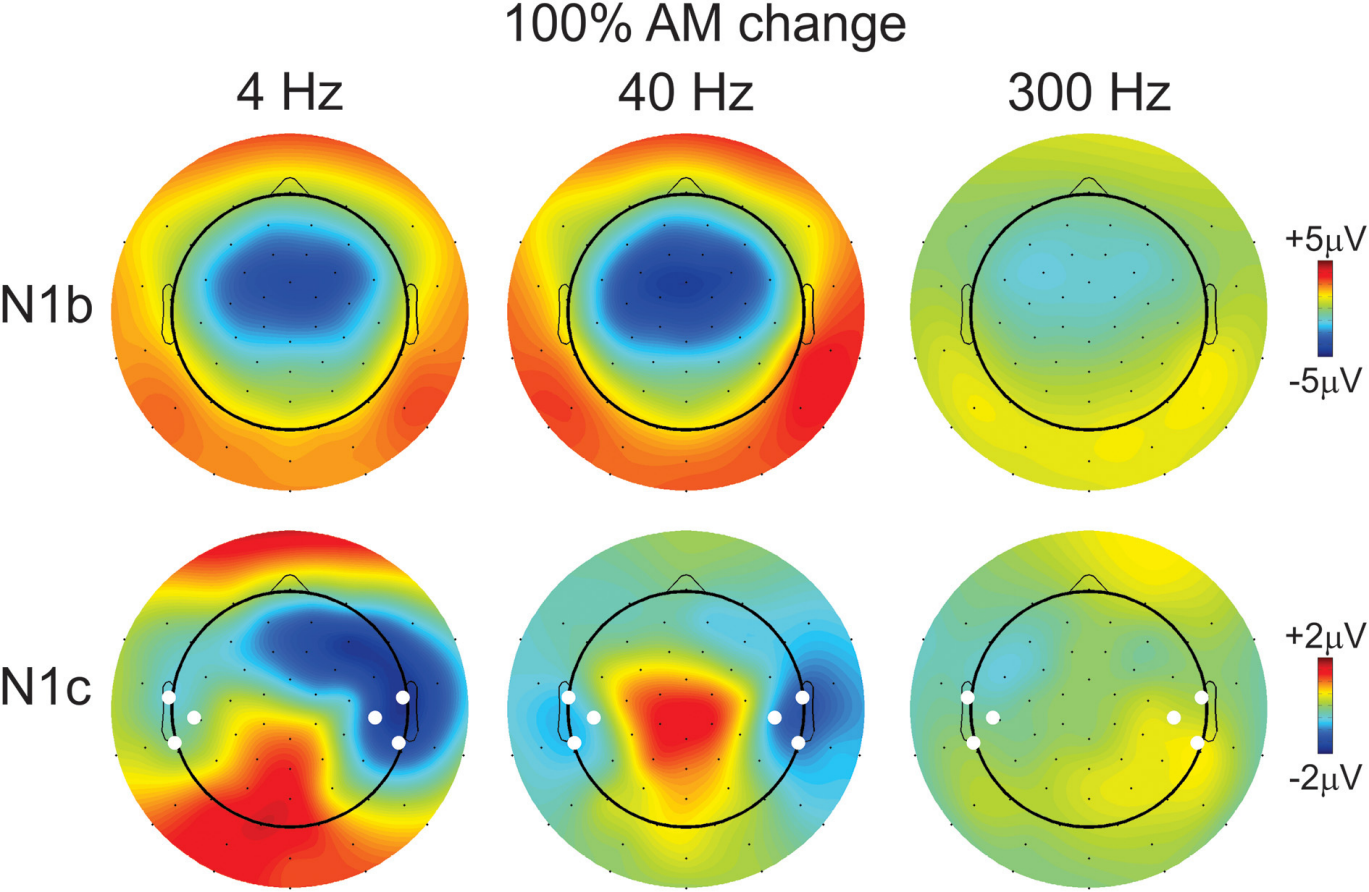
**Topography of the N1b and N1c response for the 100% AM change stimulus**. The top row shows the N1b topography for all three AM rates. The 300 Hz response is reduced compared to 4 and 40 Hz. The bottom row shows the topography of the N1c response. Note the altered distribution showing greater amplitudes (more negative) at right-sided electrodes for 4 and 40 Hz AM change stimuli. The white circles indicate which additional electrodes were pooled together to calculate a separate left- and right-sided N1c amplitude.

The effect of AM rate (Figure 3B) was examined using a repeated measures ANOVA (4, 40, 300 Hz 100% AM). For FC (N1b), there was a significant main effect for AM rate [*F*_(2, 18)_ = 26.1; *p* < 0.001]. *Post-hoc* analysis showed that N1b amplitude to 300 Hz was significantly smaller than 4 Hz (*p* < 0.001) and 40 Hz (*p* < 0.001). No difference was found between 4 and 40 Hz. For LT/RT electrodes (N1c) an interaction between RT/LT and AM rate was found [*F*_(2, 18)_ = 4.0, *p* < 0.05]. *Post-hoc* analysis revealed that N1c amplitude at RT to 4 Hz (*p* < 0.001) and 40 Hz (*p* < 0.05) were larger compared to 300 Hz. No significant difference was found between 4 and 40 Hz. Although the N1c response appeared to be larger at RT electrodes compared to LT (Figure 3) no significant difference was observed. Examination of the topography of the N1c also suggested that the 4 and 40 Hz AM change condition appeared to be greater on the right side (see Figure 4 bottom row). In order to further test the hypothesis that the right-sided N1c responses were larger than the left, additional adjacent electrodes were included in the pooled RT and LT electrodes (white circles on Figure 4). With this new pool of electrodes the right sided N1c response showed significantly greater amplitudes than the left [*F*_(2, 18)_ = 30.1; *p* < 0.001]. *Post-hoc* analysis revealed that N1c amplitude at the right hemisphere for 4 Hz were larger than left hemisphere (*p* < 0.01), no significant hemispheric differences between left and right sides were observed with 40 and 300 Hz. In our “spherical” electrode montage there are no electrodes at T3/T4 (where the N1c is typically recorded from), and because the N1c response is likely not just confined to one single electrode, as evidenced by the topography (Figure 4), we felt justified in including two additional electrodes on each side (white circles on Figure 4). A summary of the N1 measures is provided in Table 1.

**Table 1:** **Summary of mean N1b and N1c amplitudes and incidence**.

		**Depth (%)**	**N1b**		**N1c (LT)**		**N1c (RT)**	
			**Count**	**Amplitude (μV ± SD)**	**Count**	**Amplitude (μV ± SD)**	**Count**	**Amplitude (μV ± SD)**
AM change		100	10/10	−3.7 (1.7)	7/10	−0.8 (1.2)	10/10	−2.1 (0.8)
	4	50	10/10	−1.6 (0.8)	8/10	−0.3 (0.6)	9/10	−1.0 (0.6)
		25	9/10	−0.7 (0.4)	0/10	−	7/10	−0.3 (0.4)
		100	10/10	−4.4 (1.5)	9/10	−0.9 (0.7)	9/10	−1.5 (1.2)
	40	50	10/10	−2.0 (1.1)	8/10	−0.6 (0.6)	8/10	−0.6 (0.6)
		25	7/10	−0.5 (0.8)	0/10	−	0/10	−
		100	9/10	−1.2 (0.6)	7/10	−0.5 (0.6)	7/10	−0.2 (0.6)
	300	50	6/10	−0.6 (0.7)	0/10	−0.6 (0.5)	0/10	−0.4 (0.3)
		25	0/10	−	0/10	−	0/10	−
AM burst	4	100	8/10	−0.9 (1.6)	8/10	−0.7(0.7)	7/10	−0.4 (0.5)
	40	100	8/10	−1.9 (1.6)	9/10	−1.2 (0.9)	10/10	−0.9 (0.5)
	300	100	10/10	−2.2 (1.4)	9/10	−1.1 (0.7)	9/10	−0.9 (0.7)

#### AM change: effects of AM depth

**Figure 5:**
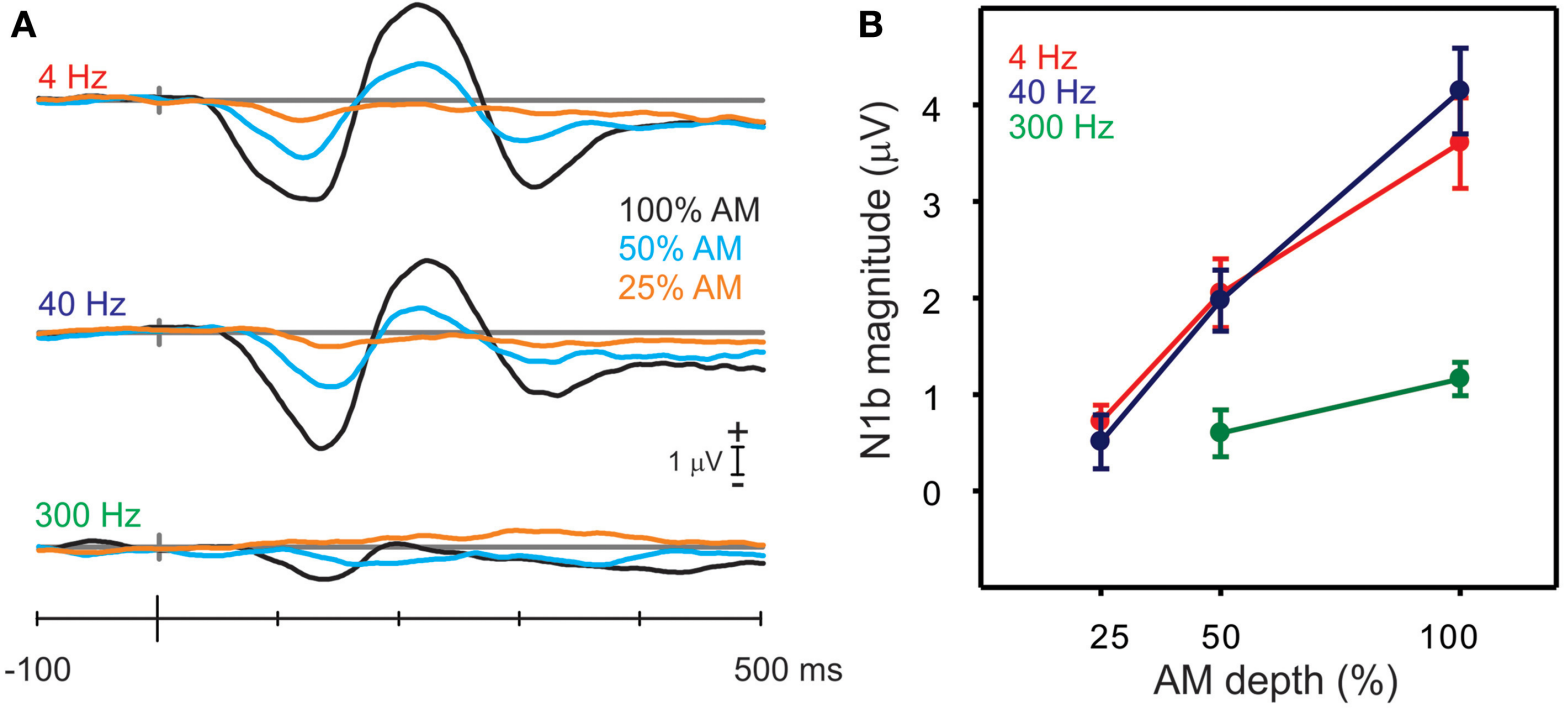
**Responses to the AM change stimuli with different AM depths**. **(A)** Shows the grand mean N1b response for all nine stimuli (3 AM rates and 3 AM depths). Note that with decreases in AM depth, there are smaller and later N1 responses. **(B)** Shows the mean amplitudes of N1b for the AM rate and depth. Note that the 300 Hz AM change response is reduced and no N1b is observed for the lowest, 25% AM depth. Errors bars: standard error of the mean.

Figure 5 shows the grand mean cortical waveforms to different AM depths. In general, N1b amplitudes decreased with smaller depths of AM.

A repeated measures ANOVA examining the effect of AM depth on N1b amplitude was performed separately for 4, 40 and 300 Hz. For 4 Hz, a significant main effect of depth was observed [*F*_(2, 18)_ = 20.9, *p* < 0.0001] such that all N1b depth amplitudes differed from each other (100% AM was larger than 50% AM *p* < 0.0001 and 50% AM was greater than 25% AM *p* = 0.003). Similarly for 40 Hz, a main effect of depth was seen [*F*_(2, 18)_ = 37.5, *p* < 0.0001], and 100% AM was greater than 50% AM (*p* < 0.0001) and 50% was greater than 25% (*p* = 0.0001). For 300 Hz, no N1b was observed for the 25% AM depth (see bottom panel Figure 5A) and therefore differences were assessed between 100 and 50% AM using a paired *t*-test and showed that 100% AM was greater than 50% AM [*t*_(9)_ = −2.49; *p* = 0.034].

The AM depth effect on N1c amplitude at RT/LT was also examined (waveforms not shown). Similar to N1b, N1c amplitudes were reduced when AM depth decreased. Repeated measures ANOVA (AM% × hemisphere) were performed for 4 and 40 Hz but not 300 Hz since no obvious N1c was apparent. For 4 Hz, a significant main effect of depth was observed [*F*_(2, 18)_ = 16.8 *p* < 0.0001] as well as a main effect of hemisphere, right bigger than left [*F*_(1, 9)_ = 10.6 *p* = 0.001]. No interactions existed would suggest that the different hemispheres had different sensitivities to AM depth. For 40 Hz, only a significant effect of AM depth was observed [*F*_(2, 18)_ = 6.25 *p* = 0.009]. As with the 4 Hz data, no interaction between hemisphere and AM depth was observed that would suggest different AM depth sensitivity across hemispheres.

#### AM change vs. AM burst

**Figure 6:**
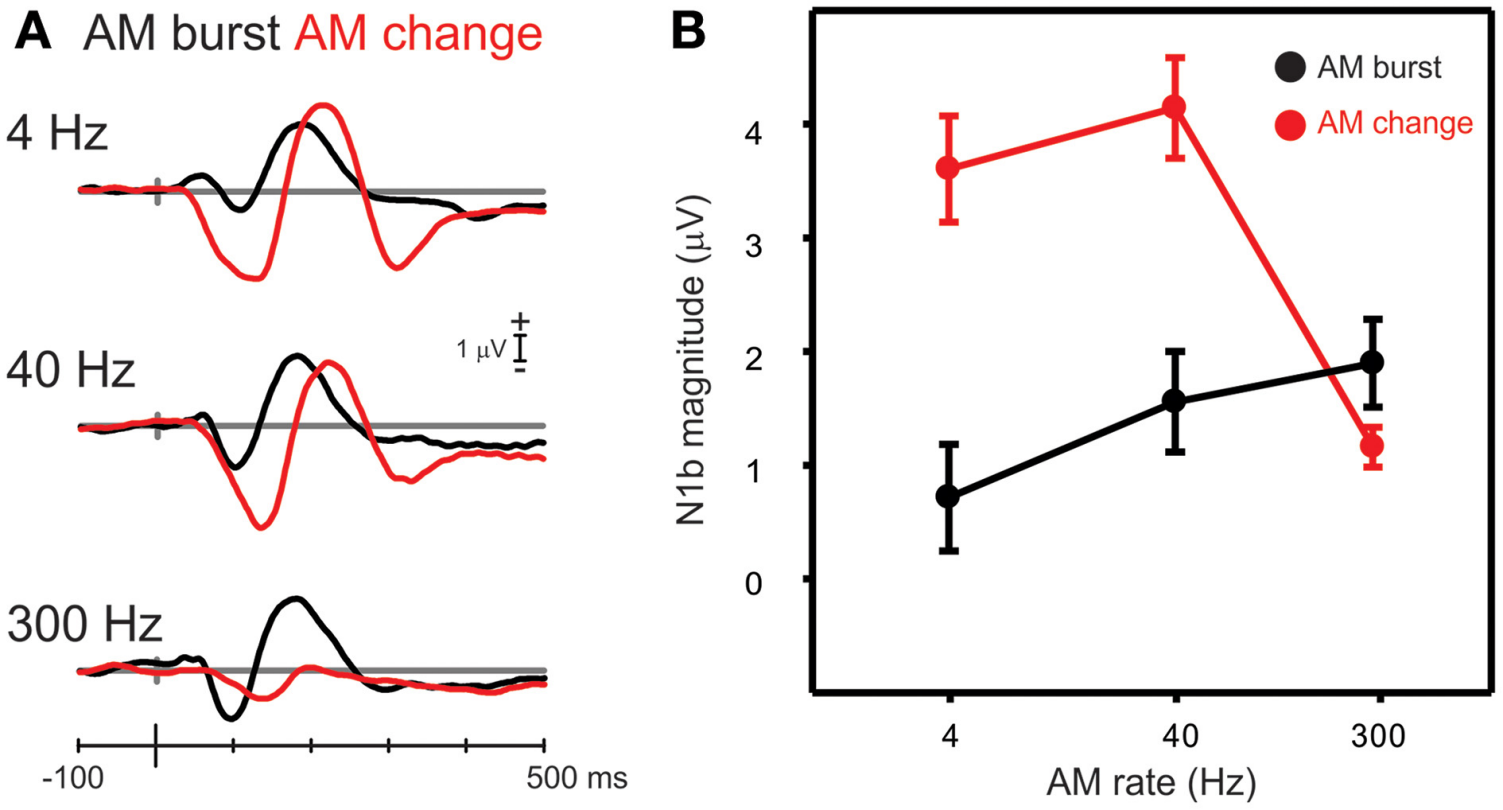
**A comparison of N1b responses to AM change with AM burst**. **(A)** Shows the grand mean waveforms while **(B)** shows the mean N1b response over a 20 ms window for all participants. Note the AM change responses were larger than the AM burst for 4 and 40 Hz AM, but not with 300 Hz. Errors bars: standard error of the mean.

Figure 6 compares the grand mean cortical waveforms to AM burst and change differed at the three AM rates (all at 100% depth). AM burst responses showed a consistent increase in amplitude with increases in AM rate, whereas AM change resembled a low pass filter shape, such that 4 and 40 Hz AM showed large responses while the 300 Hz AM change response was small. Corresponding topography plots for N1b and N1c for AM burst are shown in Figure S2. In contrast to the AM change, the N1c topographies to AM burst (Figure 4) showed no apparent hemispheric asymmetries.

Differences in N1b amplitude between AM burst and AM change were compared for each AM rate. A repeated measures ANOVA (AM rate × stimulus type) revealed a significant interaction between AM burst and AM change [*F*_(2, 18)_ = 41.0; *p* < 0.001]. *Post-hoc* analysis revealed that AM change for 4 and 40 Hz was larger than AM burst 4 and 40 Hz (both *p* < 0.001). Examination of Figure 6A suggested that there may be a latency difference between burst and change responses. Therefore, a repeated measures ANOVA was performed on the N1b latency (AM rate × stimulus type). A significant main effect was observed indicating that AM change responses were of longer latency than AM bursts [*F*_(1, 9)_ = 22.0; *p* = 0.001]. A repeated measures ANOVA (change/burst × hemisphere × AM rate) was performed in order to assess whether differences existed between N1c (left and right), 4 and 40 Hz, and AM burst vs. AM change. A significant interaction [*F*_(1, 9)_ = 8.7; *p* = 0.016] was observed and *post-hoc* analysis revealed that 4 Hz AM burst was greater than AM change for the left N1c (*p* = 0.032). Additionally, the 4 Hz AM change N1c was greater on the right side compared to the left (*p* = 0.041; data not shown).

#### Dipole source analysis

Dipole source analysis was carried out for two reasons. Firstly we wanted to examine the different relationships of tangential and radial components with AM rate. This would allow us to assess the relative contributions of primary auditory cortex (tangential source) and secondary auditory cortex (radial source) to AM rate coding. Additionally, examination of N1c topography (Figure 4) suggested that the right-sided radial source may be larger for 4 and 40 Hz AM change. The results of the individual regional dipole fits for the 100% AM conditions revealed that not all subjects met the criterion for 80% GOF. For AM burst, 4, 40, and 300 Hz, 7, 7, and 9 subjects showed 80% or greater GOFs respectively. Similarly, for AM change, 9, 9, and 8 subjects had an 80% or better GOF.

***Effects of hemispheric dipoles***. Figure S3 shows both the radial and tangential source waveforms for AM change and AM burst for 4, 40, and 300 Hz and Figure S4 shows the averaged N1 source amplitude over a 20 ms window. Although the right-sided mean source waveforms appeared to be slightly larger than the left (Figure S4), no significant differences between left and right were observed for both tangential (N1b) and radial (N1c) components. This was especially surprising for the 4 Hz AM change stimulus because based on the N1c topography (Figure 4) we would have expected that the right radial source would have been larger than the left. This finding would seem to suggest that the large asymmetric topography of N1c (Figure 4) is not the result of a radial current flow to secondary auditory areas.

***Effects of AM rate***. For AM change, in the tangential orientation, a repeated measures ANOVA (AM rate × hemisphere) showed a main effect of rate [*F*_(2, 14)_ = 9.0; *p* = 0.001; ϵ = 0.004]. *Post-hoc* analysis revealed that the 300 Hz response was smaller than both 4 and 40 Hz (*p* = 0.0194 and *p* = 0.0034). Similarly for AM burst a main effect was observed for rate [*F*_(2, 10)_ = 7.8; *p* = 0.009, ϵ = 0.024] and *post-hoc* analysis revealed that the 4 Hz response was smaller than both the 40 and 300 Hz (*p* = 0.015 and *p* = 0.018 respectively). Therefore, tangential dipole source waveforms had a similar relationship with AM rate as was found with the N1b data recorded at FC. For the radial component of the AM change response, a significant effect of rate was observed [*F*_(2, 14)_ = 11.6, *p* = 0.001, ϵ = 0.001] and *post-hoc* analysis revealed that the 300 Hz rate was smaller than both 4 and 40 Hz rates (*p* = 0.0014 and *p* = 0.0067). No differences as a function rate were observed with the radial component for the AM burst responses.

#### swLORETA source analysis

**Figure 7:**
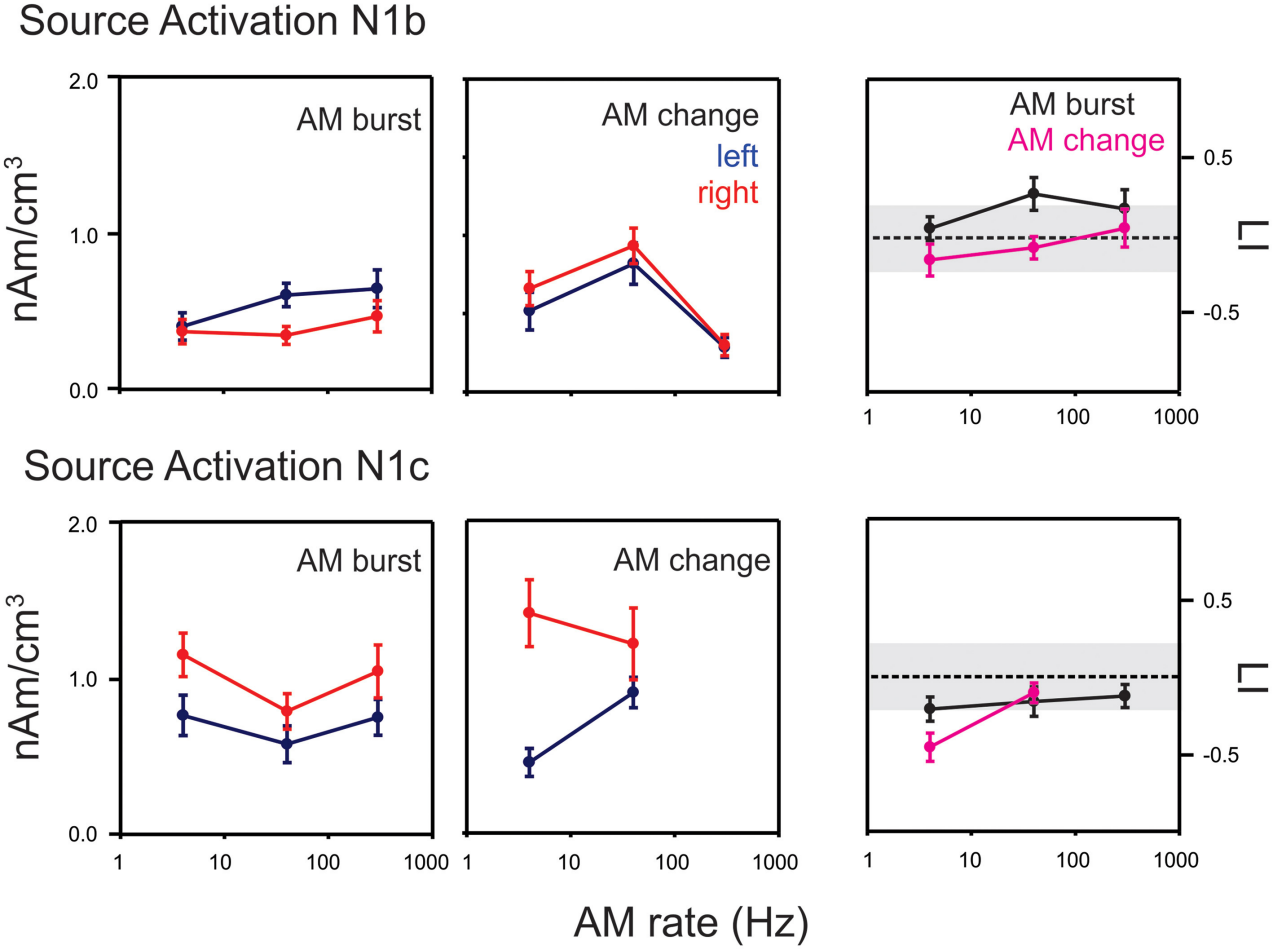
**Mean swLORETA auditory source activations for AM change and AM burst stimuli**. The top rows indicate the mean response for N1b while the bottom row shows the mean response for N1c. Responses from the left and right hemispheres are shown in blue and red respectively. The lateralization index (LI) in the right most column compares the AM change and AM burst responses. Positive: right hemisphere > left hemisphere; negative left hemisphere > right hemisphere. Most noteworthy is the finding that the AM change is larger on the right hemisphere compared to the left during the N1c time region while the AM burst is larger on the left. Gray regions of the LI plots indicate a 0.2 criterion for laterality. Errors bars: standard error of the mean.

The swLORETA approach yielded cortical activations in the auditory cortices for both AM change and AM burst. We limited our analysis to 20 ms time windows centered on N1b and N1c separately. swLORETA source images and associated virtual source time courses are shown in Figure S5. Mean amplitudes of the left and right auditory activations based on the virtual source time course for the N1b and N1c time windows are shown in Figure 7. No observable N1c component for 300 Hz AM change could be detected, therefore, the source analysis was not performed in this condition.

**Figure 8:**
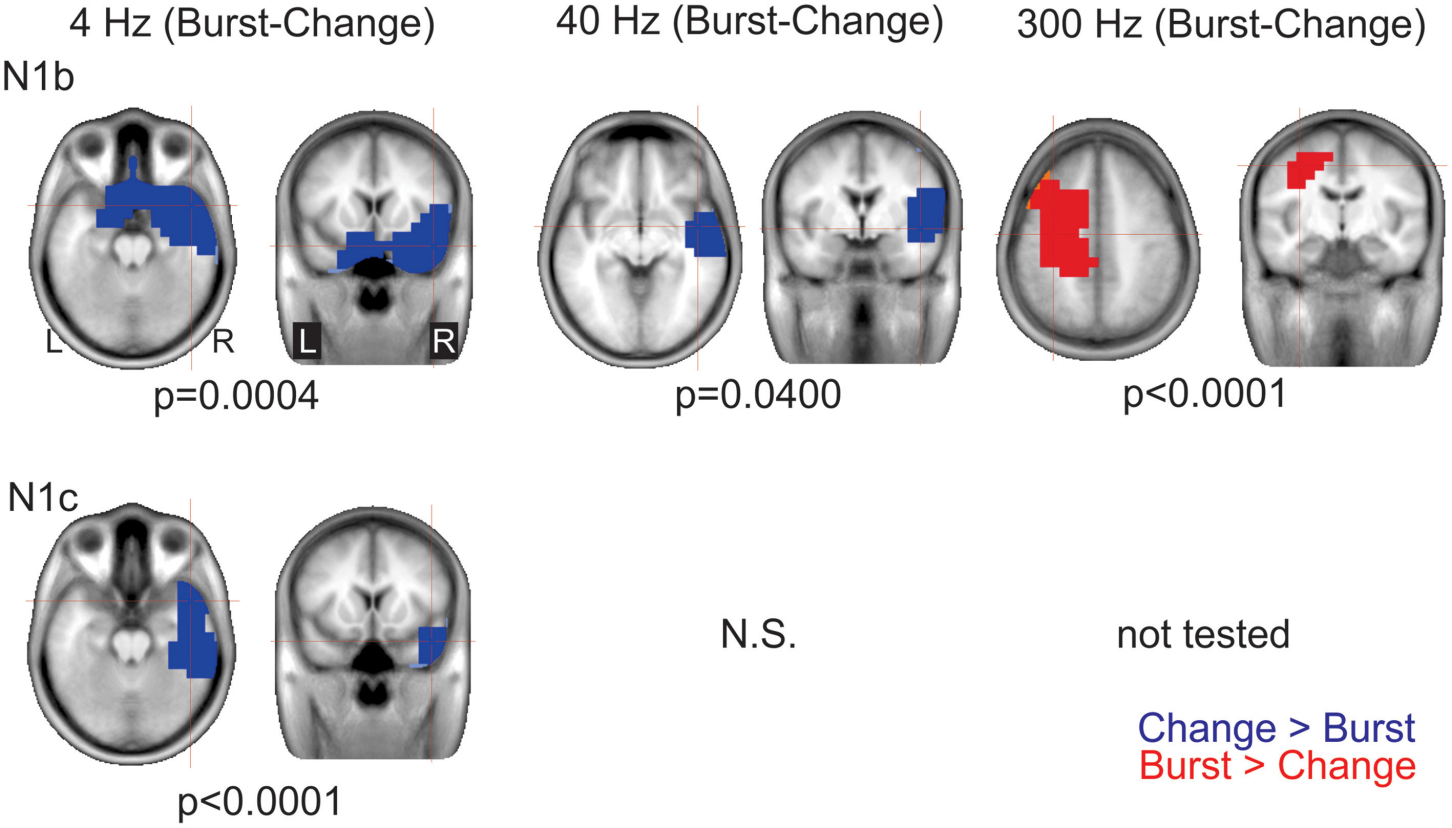
**Significant cluster differences between AM change and AM burst in source space**. The columns are organized by AM rate while the rows are organized by N1c and N1b. These data represent the difference in brain source between AM burst and AM change. If the color is blue, it indicates the AM change was greater than the AM burst (negative difference); while red indicates that AM burst was greater than AM change (positive difference). No significant (n.s.) difference was observed when the 40 Hz (N1c) change was compared to burst. Because no N1c response was observed for 300 Hz AM change, a comparison was not performed with AM burst. In general, the AM change was larger on the left auditory and frontal regions compared to the AM burst. Whereas, with 300 Hz, the burst was larger on the right. Note that these clusters indicate which regions showed a significant difference, the cross hairs indicate the 3D point which showed the maximum difference between the conditions.

For AM change, swLORETA source activations in the N1b region (top middle panel in Figure 7), a repeated measures ANOVA (AM rate × hemisphere) showed only a main effect of rate [*F*_(2, 18)_ = 17.0, *p* < 0.0001, ϵ = 0.0001], no differences were seen across hemispheres. *Post-hoc* analysis revealed that the 40 Hz response was greater than both 4 and 300 Hz responses (*p* = 0.026 and *p* < 0.001 respectively). Additionally, the 4 Hz response was greater than the 300 Hz response (*p* = 0.021). In contrast, for the AM burst (top left panel Figure 7), the activation strength did not change significantly as a function of AM rate, but rather a main hemispheric effect was observed indicating that left hemisphere activation was greater than right [*F*_(1, 9)_ = 5.7, *p* = 0.040, ϵ = 0.040]. A repeated measures ANOVA of LI for N1b (stimulus type × AM rate; top right panel Figure 7) indicated a main effect of stimulus type such that AM burst responses had a greater leftward bias compared to AM change [*F*_(1, 9)_ = 11.0, *p* = 0.009, ϵ = 0.009]. A similar analysis was performed for N1c swLORETA activations. For AM change (middle bottom panel in Figure 7) a significant interaction was observed between rate and hemisphere [*F*_(1, 9)_ = 6.6, *p* = 0.030, ϵ = 0.030]. *Post-hoc* analysis revealed that the 4 Hz response was greater in the right hemisphere than in the left (*p* = 0.0021). For AM burst (bottom left panel in Figure 7), a significant main effect of hemisphere was observed such that the overall the right activation was greater than left [*F*_(1, 9)_ = 8.9; *p* = 0.016, ϵ = 0.016]. Additionally, a main effect of rate was seen [*F*_(2, 18)_ = 4.0, *p* = 0.036, ϵ = 0.047]. *Post-hoc* analysis revealed that 4 Hz was greater than the 40 Hz (*p* = 0.0376). The LI analysis (bottom right panel in Figure 8) revealed an interaction between rate and type [AM burst/AM change; *F*_(2, 18)_ = 4.0, *p* = 0.036, ϵ = 0.036]. *Post-hoc* analysis revealed that the 4 Hz change was more lateralized to the right compared to the 4 Hz AM burst (*p* = 0.041).

***Differences in source space***. Although there a number of possible contrasts between conditions, we chose to limit our analysis to address two questions: (1) are different brain regions active during AM burst vs. AM change and (2) given that (a) the topography of the 4 Hz AM change showed a large right frontal negatively (Figure 4) and (b) that the dipole source analysis did not reveal significant hemispheric differences, and (c) that previous work has shown that the speech envelope and low rate envelopes are represented in preferentially in right auditory areas (Abrams et al., 2008; Poelmans et al., 2012) we wanted to examine the differences between 4 and 40 Hz sources, this contrast would reveal brain response differences between AM rates that exist in sentences/syllabic rate (~4 Hz) vs. faster speech elements such as formant transition (~40 Hz). Note that all *p*-values reported below are corrected for multiple comparisons.

***AM burst vs. AM change***. 4 Hz: This contrast (AM burst minus AM change) showed a significant cluster (*p* = 0.004) indicating greater right frontal and anterior temporal lobe regions with AM changes vs. AM burst N1b. A similar relationship was seen with N1c although the cluster (*p* < 0.0001) was less frontally and more laterally weighted (see Figure 8 left panels). This finding indicated that for 4 Hz, AM change stimuli activates the frontal and anterior temporal regions more than 4 Hz AM burst at both N1b and N1c latencies.

40 Hz: For N1b, AM change showed a significant cluster (*p* = 0.040) indicating greater activation in right anterior temporal lobe. No differences were seen between AM burst and AM change for N1c (see Figure 8 bottom middle panel).

300 Hz: For N1b AM burst showed a significant cluster (*p* < 0.0001) indicating larger activation superior to the temporal lobe. No analysis was performed on N1c given that 300 Hz AM change showed no discernible peak.

4 vs. 40 Hz AM burst: Although left hemisphere activation was larger than the right with the 40 Hz vs. 4 Hz, this difference was not significant.

**Figure 9:**
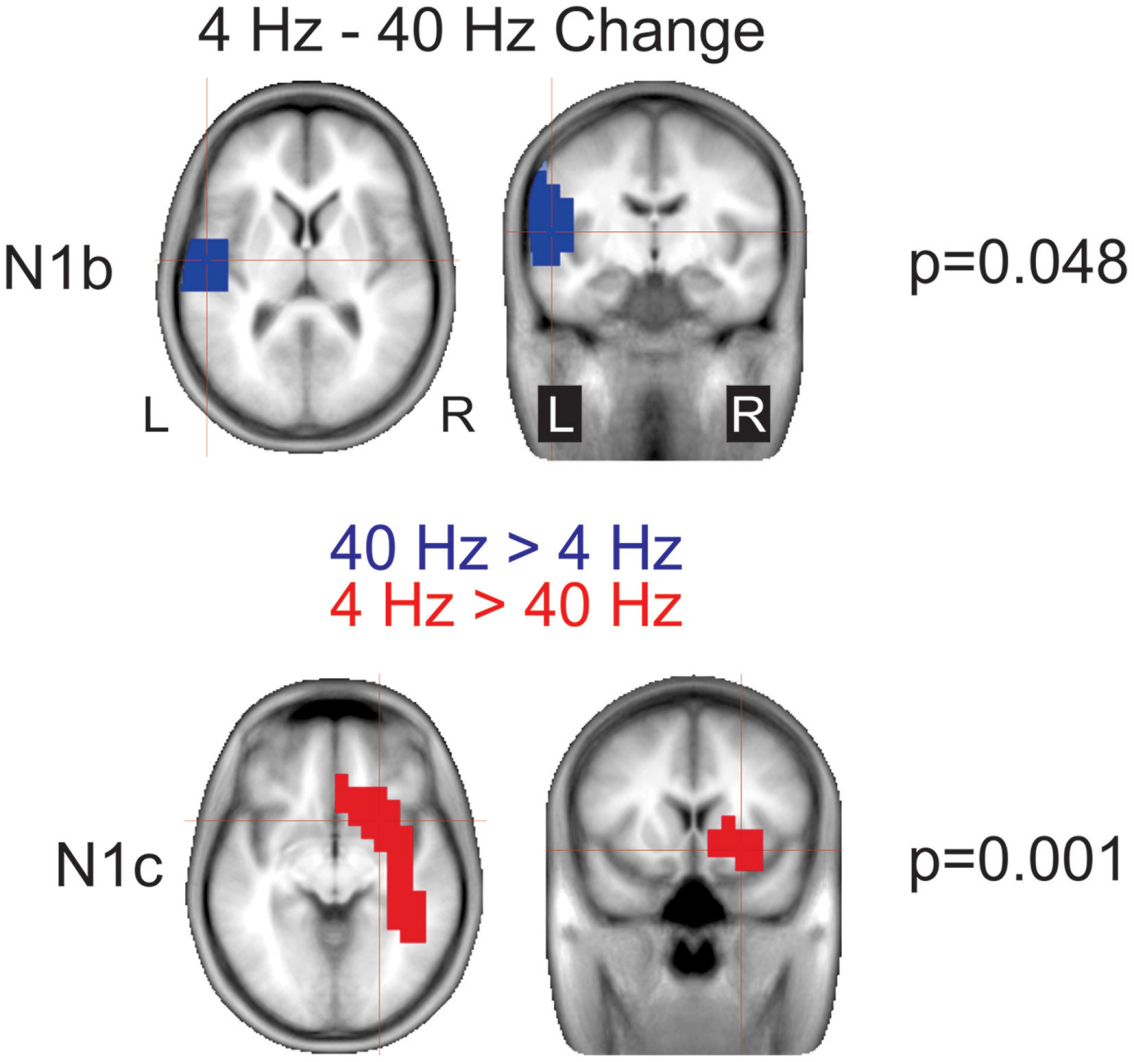
**Significant cluster differences between 4 and 40 Hz AM change**. These data represent voxels that showed a significant difference between 4 and 40 Hz. The color indicates the direction of the difference such that when the cluster is blue, or negative 40 Hz was greater than 4 Hz (4 Hz minus 40 Hz) and similarly, red indicates a positive difference such that 4 Hz is greater than 40 Hz. Note that these are significant clusters and the cross hairs are focused on the maximum difference between the contrast. Note that 40 Hz shows greater left hemisphere activation during N1b and 4 Hz shows a greater right hemisphere activation during N1c.

4 vs. 40 Hz AM change: For the N1b difference a significant cluster (*p* = 0.048) in the left temporal lobe indicated that the response to 40 Hz was larger than 4 Hz (Figure 9 top). For the N1c, a significant cluster (*p* = 0.001) was seen in the right temporal lobe extending into frontal regions indicating that the response to 4 Hz was larger than 40 Hz (Figure 9 bottom).

## Discussion

The present study of auditory cortical activity to AM change in normal-hearing listeners revealed four findings: (1) for AM change, N1 responses were robust to low AM frequencies and less so to high AM frequencies. This pattern of activity is similar to psychoacoustic TMTFs such that the AM thresholds are lower for low AM rates and higher for high AM rates. (2) Low rate AM change (4 Hz) activates right temporal and frontal regions known to be associated with speech and prosody processing. (3) There was a discordance between dipole source analysis and swLORETA suggesting that with multiple concurrent sources modeling brain activity with only two dipoles is likely an over simplification. (4) AM burst stimuli resulted in responses greater on the left hemisphere.

### AM change as a paradigm to assess cortical temporal processing

Traditionally, electrophysiological measures of temporal processing have either used ASSRs, gap detection, or paired stimuli (reviewed in Picton, 2013). The ACC does not examine the auditory system's ability to follow the envelope of the stimulus (like with ASSRs) but rather the ability to discriminate AM from unmodulated white noise. We believe that this process more closely resembles the behavioral TMTF task that a subject would be actually performing rather than the ability to phase-lock over time to the modulation.

**Figure 10:**
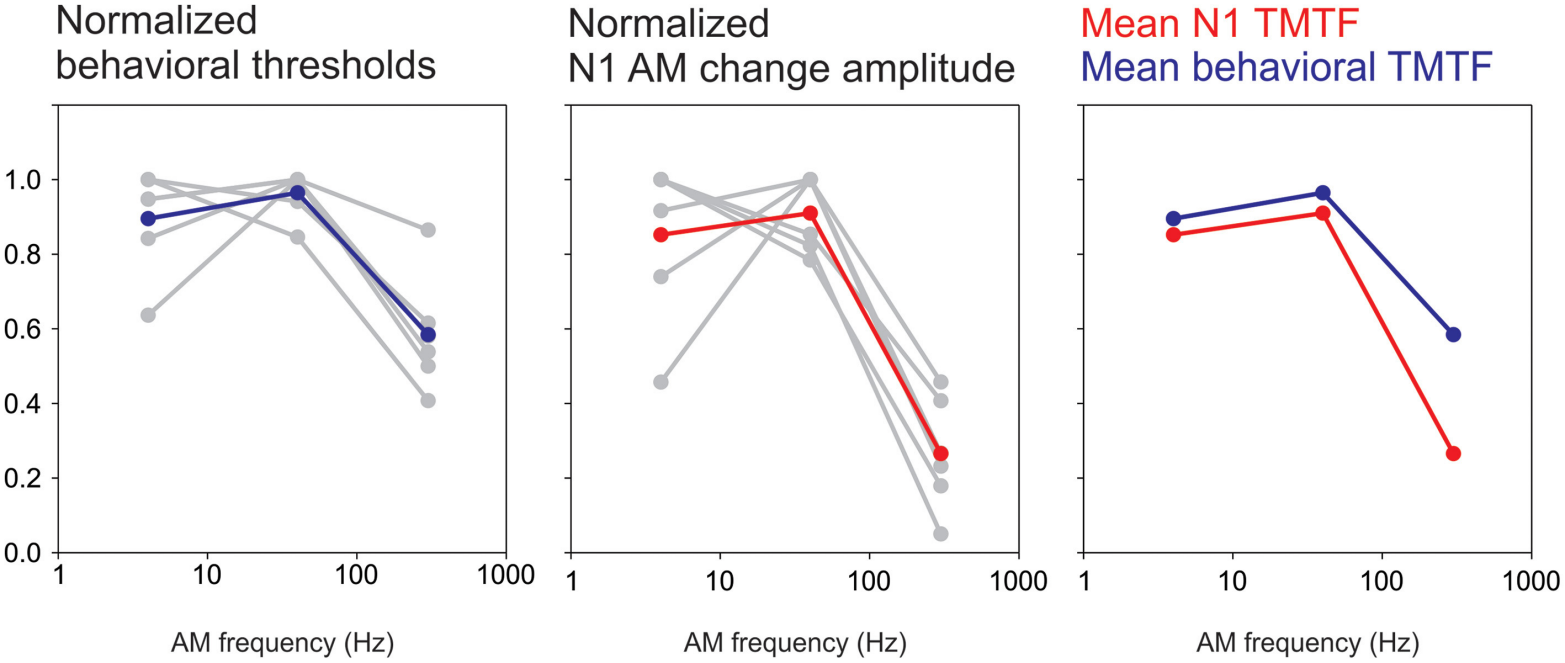
**A comparison between an N1-based TMTF and a behavioral-based TMTF**. Six subjects had both behavioral TMTFs and AM change responses. For the behavioral normalization, the smallest AM detection threshold (across the three AM rates) for each subject was used as a “reference” and all other AM depth thresholds were calculated as a ratio difference from the reference. Individual normalized behavioral AM detection thresholds are shown in gray while the mean across subjects is shown in blue. Note that for all subjects the smallest AM threshold was either 4 or 40 Hz AM. A similar process was performed for N1 amplitude except that the maximum amplitude was used a reference and all other responses (at the other AM rates) were normalized as a proportion difference from the max. The middle plot shows single subjects (gray) and mean across subjects (red). The right plot compares the mean behavioral and N1 TMTFs. Note the similarity across the two methods.

The present study demonstrated a novel application of the ACC to study temporal processing at the cortical level. Behaviorally, the TMTF is determined by finding the minimum AM depth needed for modulation detection by progressively decreasing the depth of AM until the listener can no longer detect modulation. In contrast, this study was mostly concerned with suprathreshold AM depths. We found that the N1 amplitude to AM change was robust at low AM frequencies and less sensitive at high AM frequencies exhibiting a pattern similar to behavioral TMTFs (Viemeister, 1979; Bacon and Viemeister, 1985). This paralleled the behavioral data from the attend condition showing reduced detection rates for the 300 Hz 25% AM and longer reaction times to the 300 Hz stimulus compared to 4 and 40 Hz. In the psychoacoustic literature, TMTFs are often modeled as low-pass filter functions with fitted parameters relating to overall detection level, slopes and cut off rates. Unfortunately in this study only three AM rates were used. Therefore, fitting a low-pass filter function to N1 data was problematic and made formal quantitative comparisons difficult (i.e., cut off and slope differences between N1 and behavior). Nonetheless, we attempted to compare the N1 response as a function of AM rate with the behavioral TMTF in the 6 subjects for whom we had both sets of data. In order to facilitate the comparison, we normalized both the N1b response and behavioral thresholds to a percent change. When plotted this way both N1b TMTFs and behavioral TMTFs were very similar in appearance (Figure 10). Both plots showed a maximum at 40 Hz while the 300 Hz AM response was greatly reduced. A future study will formally compare fit functions with more AM change rate stimuli.

In contrast to the AM change stimuli, the AM burst function with AM rate had an opposite shape such that large N1 responses were seen with faster AM rates and small N1 responses with low rate AM. The N1 amplitude to the AM burst is likely dominated by the rise time of the stimulus similar to the responses elicited by tone bursts (Onishi and Davis, 1968; Skinner and Jones, 1968). This was particularly evident at the two extreme AM rates, 4 Hz burst since the rise time (zero to maximum amplitude) is 125 ms (1/2 cycle of AM) whereas for 300 Hz it is 1.6 ms. Therefore, this would suggest that electrophysiological paradigms involving onset burst type stimuli would likely not be related to behavioral TMTFs. Although, it may be conceivable to use “standard” AM bursts as with different AM rates as deviants in a mismatch negativity paradigm.

In this experiment, we varied the AM depth in large discrete steps (100%, 50, and 25%) and therefore this approach differs compared to an adaptive threshold seeking paradigms typically used in psychoacoustic studies. Although it is possible to vary the AM depth in smaller increments to determine minimum AM depths needed to elicit an N1, such an approach would likely require much longer recording times. Nonetheless, at smaller AM depths, the N1 response decreased in magnitude and was absent when barely detectable (9% for 300 Hz, 25% depth).

Interestingly, the behavioral detection of AM in continuous white noise is different from what is typically measured behavioral in TMTF studies. For high AM frequencies (near 300 Hz) the AM detection is typically near 20% (~-14 dB) which is similar to what was observed in the six subjects (25%) who performed the standard TMTF behavioral paradigm. However, even in these same subjects, performance in the attend condition was greatly reduced to such a degree that most subjects could not even detect the 300 Hz 25% AM change stimulus (see Figure 2A). For example in these 6 subjects the mean detection rate for the 300 Hz, 25% AM stimulus was 12% (based on 100 trials), whereas their mean AM detection threshold on the three alternative forced choice task was 25%. This is likely related to the nature of the attend condition paradigm. We instructed subjects to detect *any* change that could include 4, 40, or 300 Hz at 100, 50, 25, or 0% AM depths and therefore subjects needed to be “broadly AM tuned” in the attend condition rather than be focused on one particular AM rate as is the case during a standard TMTF task. This phenomenon may be related broad “listening bandwidths” observed with frequency discrimination (Schlauch and Hafter, 1991). Similarly, when performing a frequency discrimination task, larger frequency difference limens were observed when the standard frequency was roved vs. fixed levels indicating that broad listening reduces performance (Amitay et al., 2005).

### Hemispheric asymmetries with N1b, N1c, AM burst and AM change

Differences were observed at both scalp electrode levels and in source space for both AM change and AM burst responses. At the scalp electrode level, 4 Hz 100% AM change was greater on the right compared to the left during the N1c time region (Figure 4, bottom left). At the source space level, more differences were observed and can be summarized as follows: (1) N1b to AM bursts showed larger left hemispheric activation than right compared to AM change (Figure 7, top left and top right); (2) all N1c responses were larger on the right hemisphere compared to the left (Figure 7, bottom panels); (3) with 4 Hz AM change, the N1c right-ward (LI) bias was greater than AM burst (Figure 7 bottom middle and right); (4) Change responses showed more right hemispheric activation compared to the left (Figure 8, top left, top middle and bottom left); (5) 40 Hz AM change activated the left hemisphere more than the 4 Hz AM change (Figure 9, top panel); (6) 4 Hz AM change activated the right hemisphere more than 40 Hz AM change (Figure 9, bottom panel).

Typically studies examining left vs. right hemispheric differences have demonstrated that hemispheric effects can be accounted through ear of stimulation effects. However, these effects are often dependent on the particular N1 component in question. N1b is maximal at the vertex with a latency near 100 ms and is thought to be generated on the superior surface of the temporal lobe in or near auditory cortex whereas N1c, also referred to as the T-complex, is maximal at temporal sites with a latency close to 150 ms and has dipole generators located on lateral surfaces of temporal lobes representing secondary or association areas of the auditory cortex (McCallum and Curry, 1979; Näätänen and Picton, 1987; Scherg et al., 1989). Differences in N1b across hemispheres have often been attributed to a contralateral dominance of the ear stimulated (Pantev et al., 1998; Picton et al., 1999). More recently, Hine and Debener (2007) also confirmed the contralateral dominance, however, difference between the hemispheres was larger with sounds presented to the right ear. The contralateral dominance of the N1c (or radial component) is less clear. Both Hine and Debener (2007) and Picton et al. (1999) found that the radial component was larger contralaterally only with left ear stimulation. In the current study however, we did not anticipate finding these types of ear-related asymmetries because all of the sounds were presented from a single loud speaker directly in front of the subject. Therefore, any hemispheric asymmetries observed would be attributed to specialized hemispheric acoustic processing rather than be driven by the ear of stimulation.

Onset vs. change: In our study we found that N1 ACC responses were larger and more delayed compared to N1 onset responses (Figure 6). Additionally, the ACC responses were more anterior compared to onset responses (Figure 8). These two observations suggest that change and onset are processed differently in the cortex. Part of this difference is related to the different rise times as mentioned above. However, another aspect may be related to how the brain encodes *change* differently than onsets. Previous work that has examined change vs. onset responses have found varied effects on both the source of the evoked potential (anterior vs. posterior), the specific evoked potential component (N1b vs. N1c/T-complex) and left vs. right hemispheres. Using a pitch and timbre change paradigm involving musical notes, Jones et al. (1998) found that change responses were more posterior compared to onsets and the N1c response was greater on the right hemisphere. No differences in source localization were found using frequency change vs. bursts of tones in a study by Yamashiro et al. (2011). Krumbholz et al. (2003) examined a change response, (referred to as pitch onset response; POR) that was generated in response to a white noise to ripple noise (pitch percept) transition and found that the POR has a more anterior source compared to an onset N1, a similar anterior POR has also been shown by others (Gutschalk et al., 2004; Bidelman and Grall, 2014). In a follow up study, Seither-Preisler et al. (2006) found no hemisphere difference with the POR. Other studies examining change responses to vowel and noise transitions found both anterior and posterior differences compared to onset responses that was stimulus transition dependent (Edmonds et al., 2010). In another change paradigm using binaural noise eliciting a Huggins pitch, Chait et al. (2006) did not report a anterior-posterior difference but rather a left hemisphere dominance. Some authors have also suggested that “change responses” are related to the mismatch negativity (MMN) since both responses are indexed by change in an ongoing sequence (Lavikainen et al., 1995). In a continuous tonal frequency change paradigm, Lavikainen et al. (1995) noted that the change elicited a double peaked N1. When modeled with two dipoles, the first peak was attributed to a normal onset response and the other, labeled as a MMN component, had a more anterior source. Our ACC data to 4 and 40 Hz also had a more anterior source compared to the AM bursts similar to a MMN (see Figure 8) and may be related to the frontal source of the MMN (see review on MMN frontal sources recently reviewed Deouell, 2007) but also encompasses anterior portions of the superior temporal sulcus. Along similar lines (Jääskeläinen et al., 2004) used a N1 adaptation paradigm to tones of different frequencies to investigate the generation of the MMN. They suggested that an early N1 (~85 ms) with broad tuning was followed by a more anterior component of N1 generated by unadapted, feature specific neurons. Therefore, our results of a more anterior generator for the ACC suggest that similar cortical areas are recruited as the MMN or unadapted neurons in novelty detection. All these “change” studies highlight the general observation that change responses are generated by different regions of cortex than the control onset responses, how they differ are likely related to stimulus attributes of the change itself and reflect specific feature extracting processes in cortex. In our case, the ACC response differed from onset responses by being located more anteriorly while left-right hemipshere differences appeared to be driven by the modulation rate of the AM (see below).

Hemispheric asymmetricies with the ACC: Two complimentary views of hemispheric acoustic processing are that the left and right hemispheres preferentially process temporal and spectral variation respectively (Zatorre and Belin, 2001). In this respect, a number of cortical evoked potential ACC studies have demonstrated that spectral variation preferentially activates the right hemisphere more than the left (Dimitrijevic et al., 2008; Okamoto et al., 2009) and temporal variation preferentially activates the left more than the right (Okamoto et al., 2009). Another common interpretation of the hemispheric differences of acoustic processing is that the two hemispheres process sounds at different time scales. Poeppel's AST hypothesis suggests that the left hemisphere preferentially processing information at rapid time scales (~20–40 ms/25–50 Hz) while the right hemisphere extracts information at slower time scales (150–250 ms/4–7 Hz) (Poeppel, 2003). The results of this study are in general agreement with the AST hypothesis and demonstrate that the left hemisphere is preferentially activated by fast phonemic AM envelope rates (40 Hz) and the right hemisphere is preferentially activated by syllabic AM envelope rates (4 Hz). Low rate envelopes have been shown to preferentially activate the right hemisphere across a number of imaging modalities and stimulus paradigms. For example, low rate ASSRs have been shown to be more robust in the right hemisphere compared to the left (Poelmans et al., 2012; Wang et al., 2012).

N1c response amplitude showed an asymmetry that was specific to the stimulus type. For 4 Hz AM change, the right N1c was significantly larger than the left N1c, an effect not observed with AM burst. Larger right vs. left N1c responses have been previously reported (Wolpaw and Penry, 1975; Cacace et al., 1988) but not consistently in other reports (Scherg and Von Cramon, 1985). Jones et al. (1998) found larger N1c over the right temporal electrodes with the ACC to changes in timbre and pitch. These results were interpreted as a right-sided dominance for music and spectral cues. More recently, a similar finding was observed showing a greater responses on right temporal electrodes compared to left temporal electrodes in an ACC paradigm with pitch and melody changes (Itoh et al., 2012). The N1c has often been attributed to a radially orientated dipole in the 140 ms range (Scherg et al., 1989). A study by Hine and Debener (2007) showed that the radial component was larger in the right hemisphere only when stimuli were presented to the left ear, but not to the right ear, a similar finding was also found by Picton et al. (1999). Given the asymmetric topography of the 4 Hz AM change response (Figure 4) we anticipated that the 4 Hz AM change radial dipole would be larger in the right hemisphere compared to the left. This was not the case (Figure S4, bottom left) and we therefore conclude that the N1c asymmetry with 4 Hz AM change is likely not due to a difference in radial component strength but rather another more anterior generator with an overlapping time course, in effect causing an apparently larger N1c. Evidence of this anterior source can be seen in the statistical brain voxel maps seen in Figure 8 (left panels) comparing 4 Hz AM burst and AM change. This data suggests that right anterior regions are significantly selectively activated during the AM change compared AM burst. The discrepancy between the dipole source analysis and swLORETA is likely related to multiple source activations that are not appropriately modeled with single dipoles. A similar discussion regarding the discrepancies between dipoles and distributed sources was recently addressed in a paper examining the effects of directed attention on ASSRs (Bharadwaj et al., 2014). Similar right anterior activations were observed in Zatorre and Belin (2001) and Jamison et al. (2006) in auditory spectral listening paradigms and therefore these data provide additional evidence that the right hemisphere is more activated than the left for processing low frequency envelopes typically found in speech (Abrams et al., 2008). The contrast between 4 and 40 Hz AM change during N1c (Figure 9) demonstrate that right anterior regions are preferentially active for low vs. high rate AM envelopes also suggesting that this region is preferentially activated for syllable-like stimulus durations. During the N1b time region, the 4–40 Hz contrast showed greater activation on the left hemisphere for fast AM processing (Figure 9 top). For 4 Hz AM change, there was a symmetrical N1b response indicating that at this stage of neural processing, cortex has still processed the stimulus, but the activity does not differ across hemispheres.

### Clinical applications

We confirmed that cortical N1 responses evoked by AM change showed similar patterns to behavioral TMTFs. The relationship between electrophysiological measures and behavioral measures may provide insight into the development of an objective measure of speech perception for various clinical populations with temporal processing deficits, such as sensorineural hearing loss, auditory processing disorder, and auditory neuropathy. Clinically, early detection of these conditions is critical for early intervention and normal speech development. Given that current clinical diagnosis for these conditions depends on behavioral responses from the subjects, it is critical to develop an objective tool to identify the site of dysfunction in the auditory pathway and relate these sites to speech perception outcome. Current electrophysiological methods utilizing simple clicks, tones, or speech sounds have shown poor correlation with speech perception (Abbas and Brown, 1991). Part of the reason of the poor relationship with speech perception is that often these measures focus on obtaining pure tone thresholds rather than more ecologically valid suprathreshold measures. For example, in auditory neuropathy there is a poor relationship between the pure tone audiogram and speech perception (Starr et al., 1996), and research focused on assessing suprathreshold measures such as gap detection (Michalewski et al., 2009; He et al., 2013) or spectral processing (Dimitrijevic et al., 2008) have shown significant relationships with speech perception outcome.

### Conflict of interest statement

The authors declare that the research was conducted in the absence of any commercial or financial relationships that could be construed as a potential conflict of interest.
