# Supplementary material for: Acoustic change responses to amplitude modulation: a method to quantify cortical temporal processing and hemispheric asymmetry
Source: Front Neurosci. 2015 Feb 11;9:38. doi: 10.3389/fnins.2015.00038 (PMC4324071; doi:10.3389/fnins.2015.00038)
Supplement: Supplementary file 1 [file DataSheet1.DOCX]

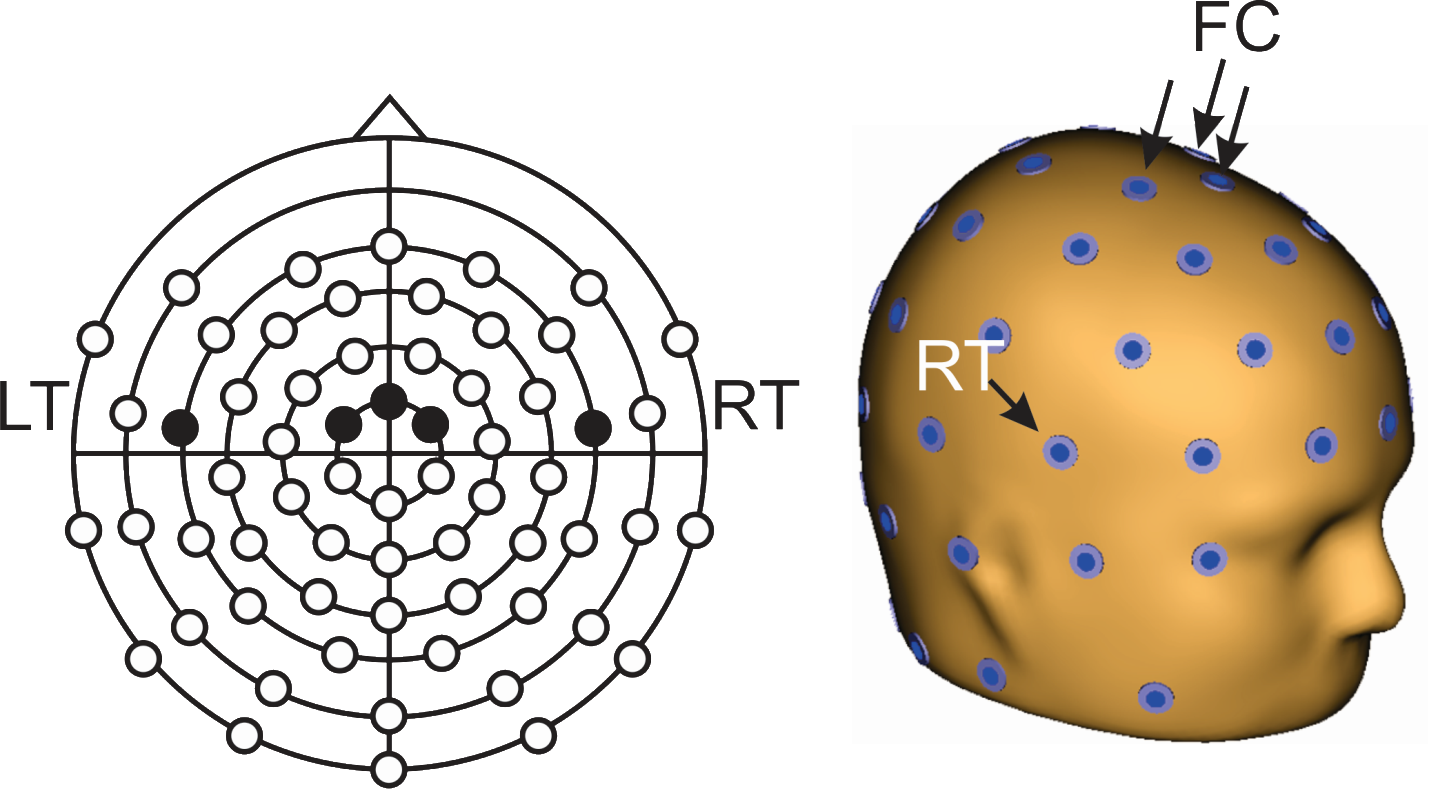


S1. The equidistant 64-channel electrode montage that was used. Indicated are the electrodes that were used in the pooled responses of FC (three arrows) and LT/RT (single arrow on RT).


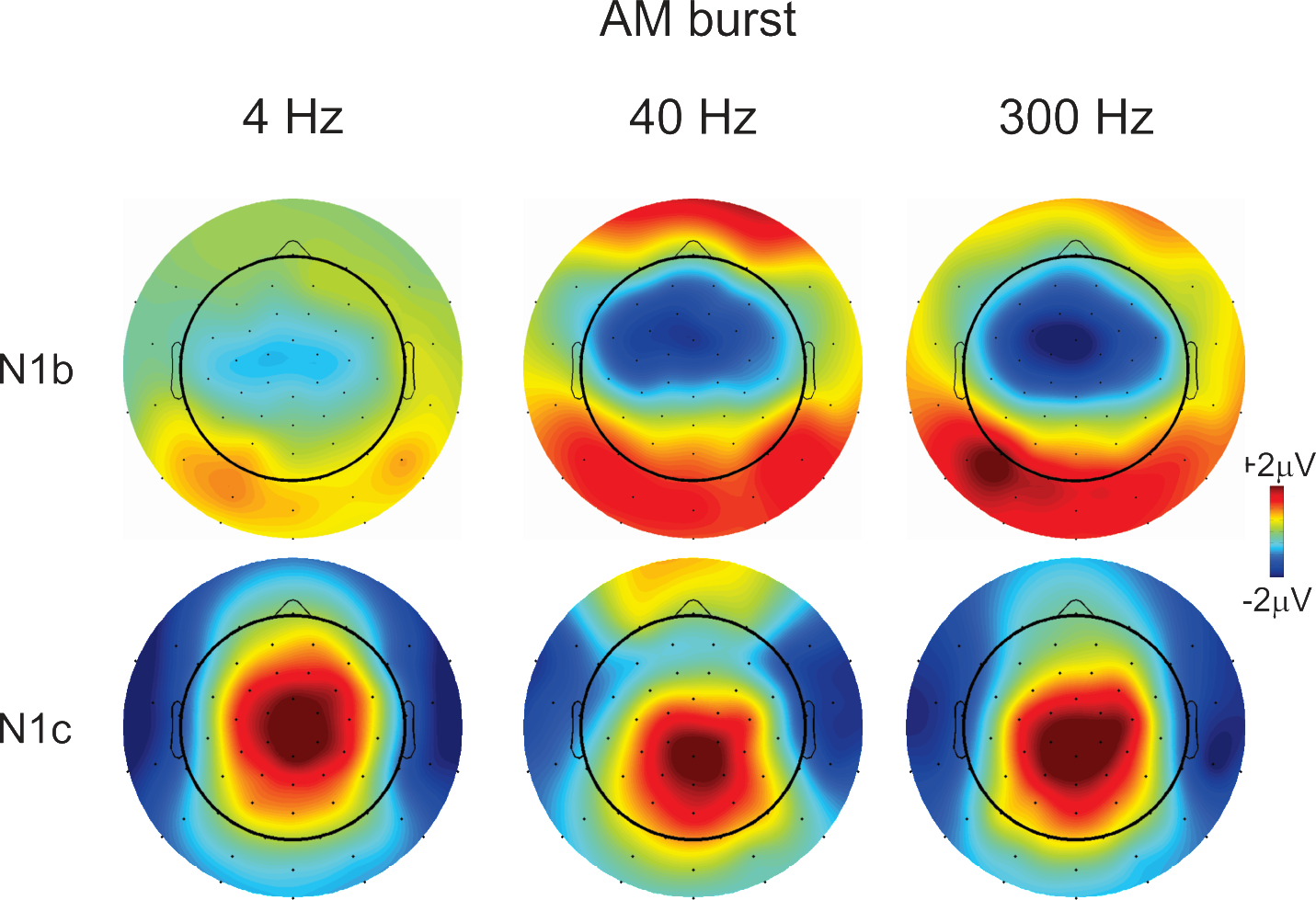


S2. Scalp topography of the AM burst responses. Tope row indicates the N1b while the bottom N1c


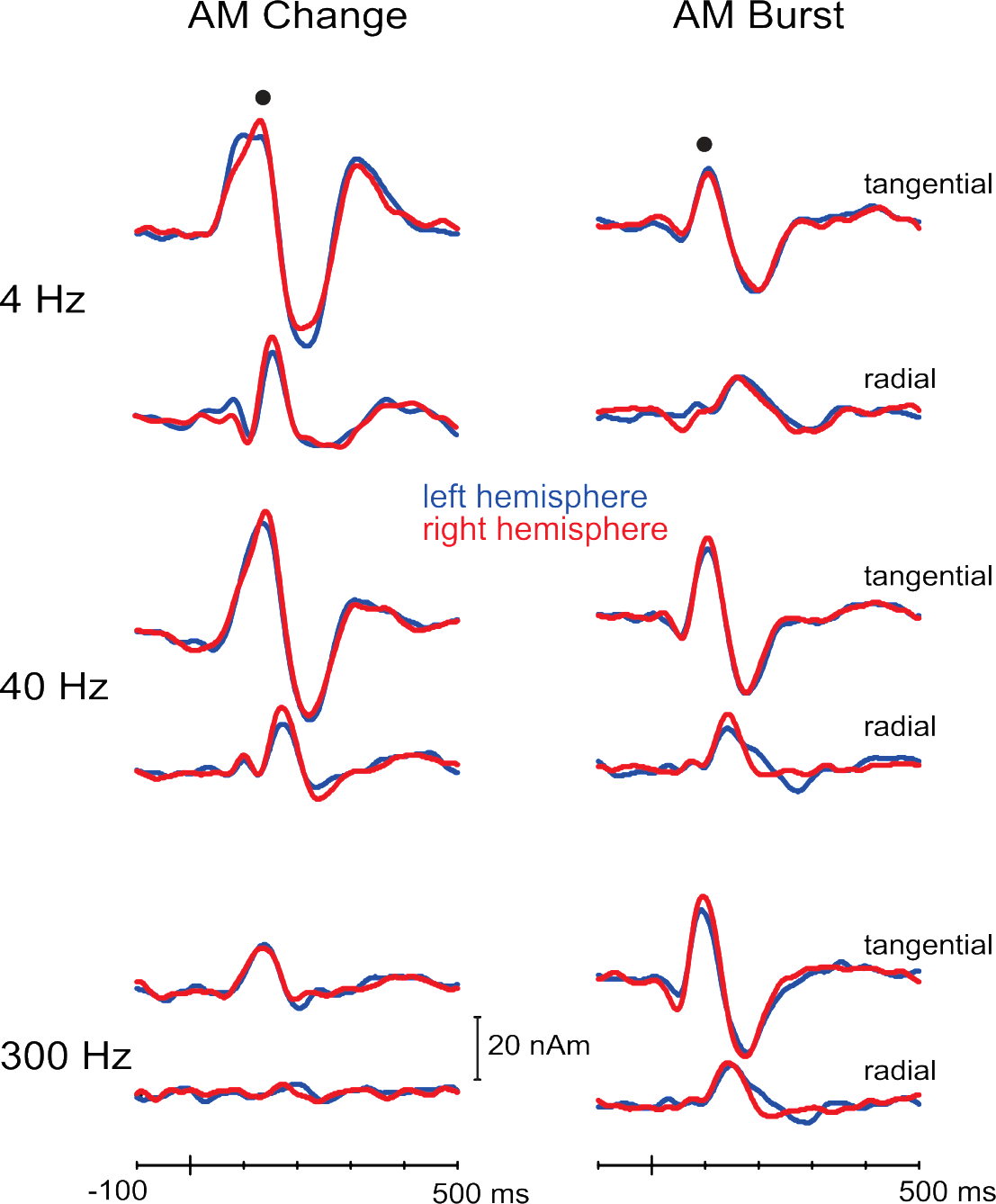


S3. A comparison of the dipole source waveforms for AM change and AM burst. Waveforms are organized into tangential and radial orientations. The N1 response is plotted upwards. The circle indicates the N1 peak. Note that no hemispeheric difference is apparent.


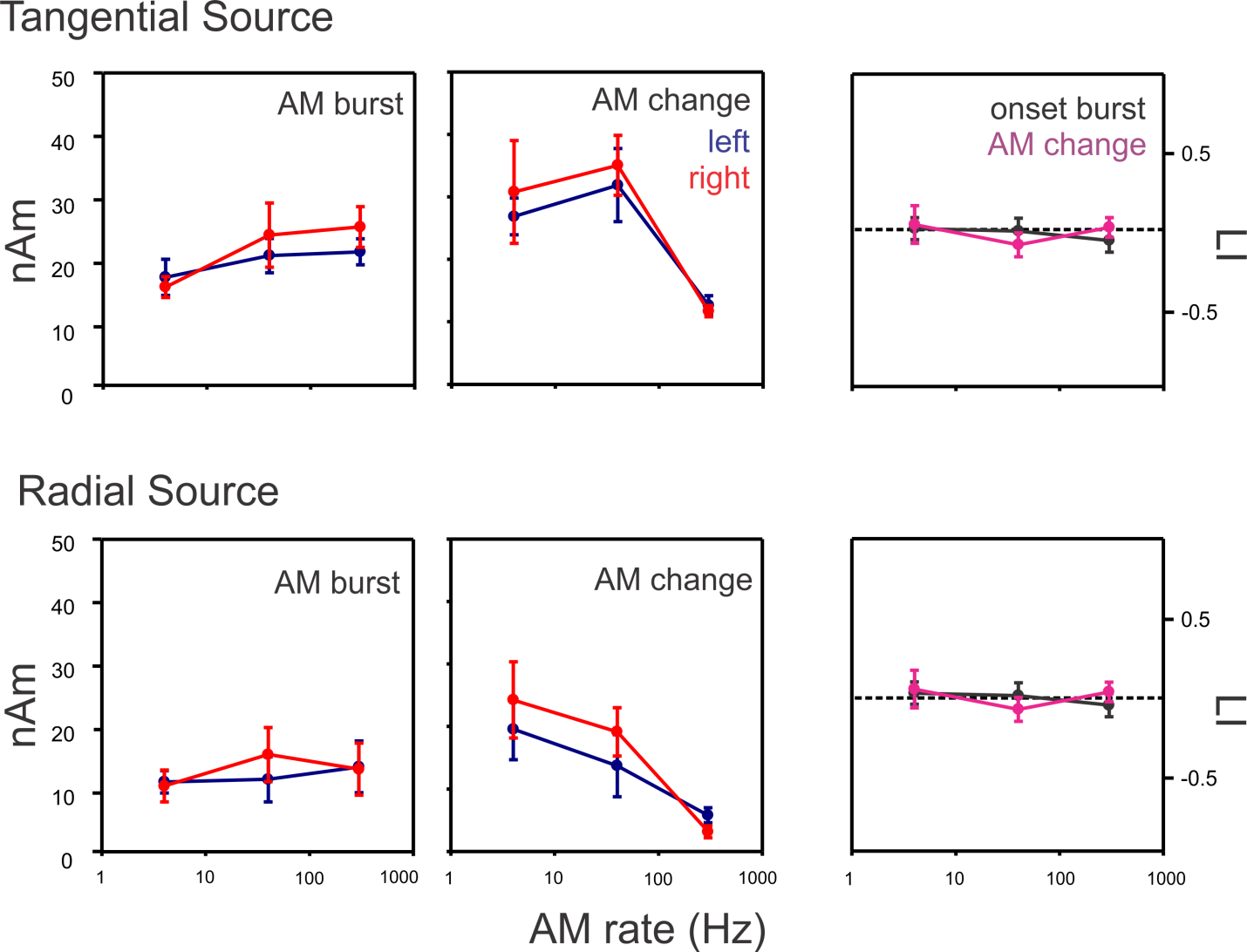


S4. A summary of the mean dipole tangential and radial sources. Left and right hemispheres are shown in blue and red. The lateralization index (LI) is shown in the right most column. Although the AM change radial source appeared to be slightly larger on the right, this difference was not significant.


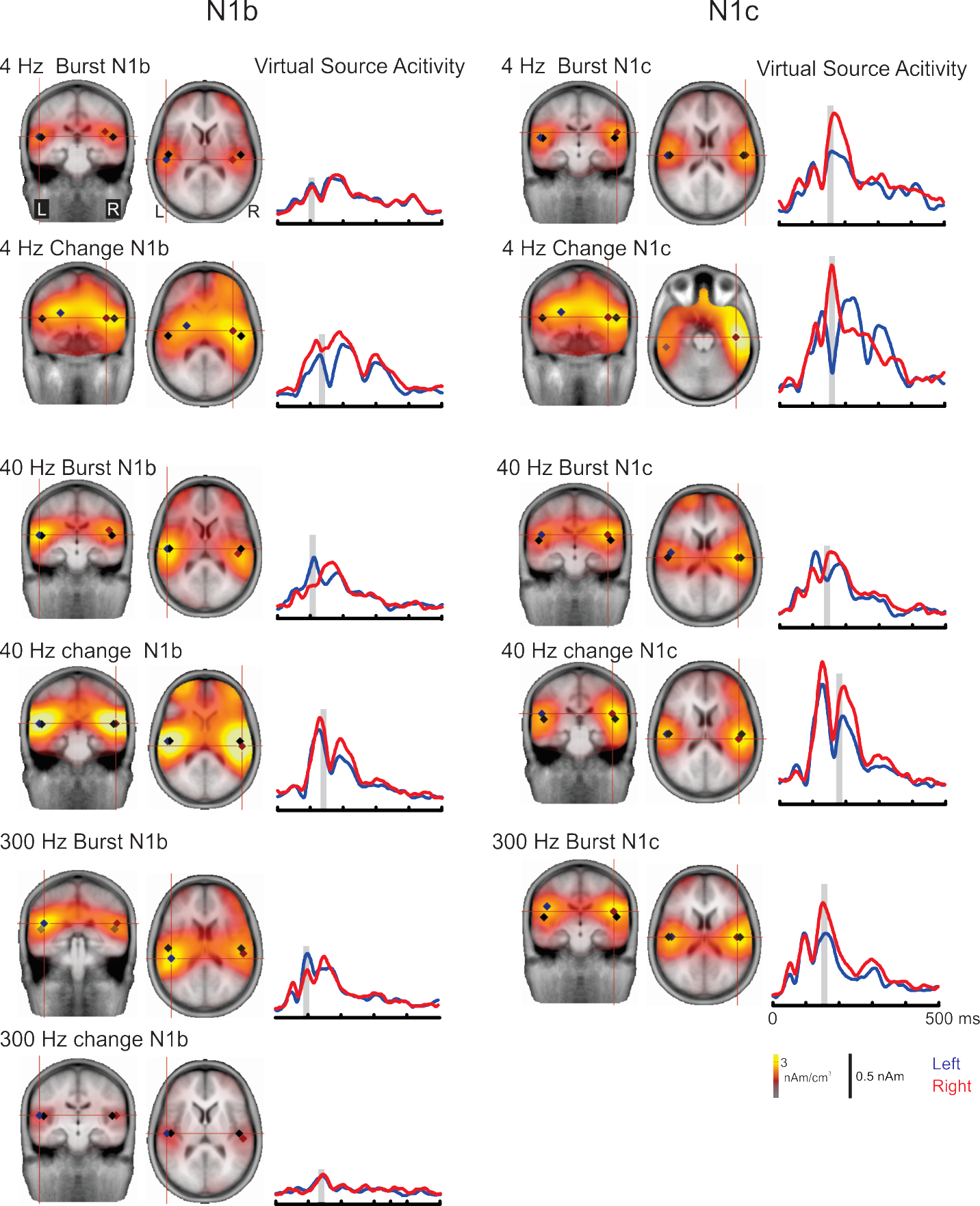


S5. swLOREA activations are shown. The left and right columns show N1b and N1c activations. Virtual source activity is shown for left (blue) and right (red) hemispheres. The particular voxel chosen for the source activity was based on the maximum activation (from the grand mean swLORETA) for each AM rate separately for N1c and N1b. The red and blue diamonds indicate the voxel location of the maxim right and left hemisphere activation respectively. The grey bars indicate the time window for each particular N1b/N1c maximum. The black diamonds are placed near Heschl’s gyrus Talairch coordinates (±49.5, -17, 9).
